# Population genetic analysis of a global collection of *Fragaria vesca* using microsatellite markers

**DOI:** 10.1371/journal.pone.0183384

**Published:** 2017-08-30

**Authors:** Hrannar Smári Hilmarsson, Timo Hytönen, Sachiko Isobe, Magnus Göransson, Tuomas Toivainen, Jón Hallsteinn Hallsson

**Affiliations:** 1 Faculty of Agricultural and Environmental Sciences, Agricultural University of Iceland, Keldnaholt, Reykjavik, Iceland; 2 Department of Agricultural Sciences, Viikki Plant Science Centre, University of Helsinki, Helsinki, Finland; 3 Department of Biosciences, Viikki Plant Science Centre, University of Helsinki, Helsinki, Finland; 4 Kazusa DNA Research Institute (KDRI), Kisarazu, Chiba, Japan; 5 Department of Plant Sciences, Norwegian University of Life Sciences, Ås, Norway; USDA-ARS Southern Regional Research Center, UNITED STATES

## Abstract

The woodland strawberry, *Fragaria vesca*, holds great promise as a model organism. It not only represents the important Rosaceae family that includes economically important species such as apples, pears, peaches and roses, but it also complements the well-known model organism *Arabidopsis thaliana* in key areas such as perennial life cycle and the development of fleshy fruit. Analysis of wild populations of *A*. *thaliana* has shed light on several important developmental pathways controlling, for example, flowering time and plant growth, suggesting that a similar approach using *F*. *vesca* might add to our understanding on the development of rosaceous species and perennials in general. As a first step, 298 *F*. *vesca* plants were analyzed using microsatellite markers with the primary aim of analyzing population structure and distribution of genetic diversity. Of the 68 markers tested, 56 were polymorphic, with an average of 4.46 alleles per locus. Our analysis partly confirms previous classification of *F*. *vesca* subspecies in North America and suggests two groups within the subsp. *bracteata*. In addition, *F*. *vesca* subsp. *vesca* forms a single global population with evidence that the Icelandic group is a separate cluster from the main Eurasian population.

## Introduction

**Fig 1:**
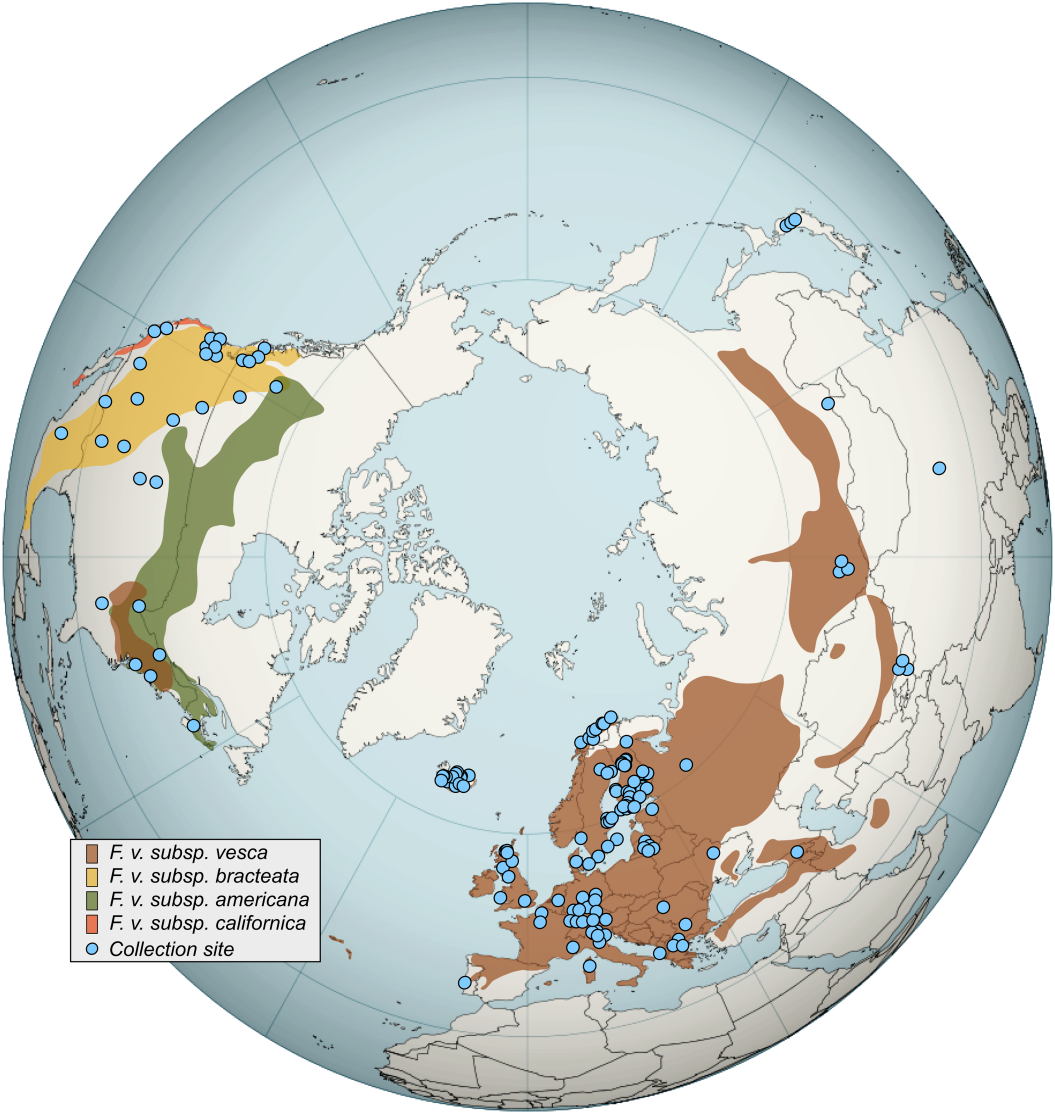
The natural geographical distribution of *Fragaria vesca* in the northern hemisphere and an overview of collection sites. Included are subspecies *F*. *vesca* subsp. *bracteata* (yellow shading), *F*. *vesca* subsp. *vesca* (brown shading), *F*. *vesca* subsp. *californica* (orange shading), and *F*. *vesca* subsp. *americana* (green shading). See supplementary S1 Table for detailed information on collection sites including coordinates. A single collection site in Bolivia is excluded from the map. Based on a map created by David Eccles, username 'Gringer', who has released this work into the public domain without any conditions. The map is available here: https://commons.wikimedia.org/wiki/File:Worldmap_northern.svg.

All species of *Fragaria* are area-specific or continentally endemic, apart from *F*. *chiloensis* and the woodland strawberry, *Fragaria vesca* L. (2n = 2x = 14). *F*. *vesca* has a vast natural distribution throughout the Holarctic [1–4] (Fig 1), with the notable exception of the North Atlantic islands of Greenland [5] and the Faroe Islands, as well as Svalbard where it has so far not been found [6]. On the other hand, *F*. *vesca* is widespread in Iceland [7–9], where it can be found on south-facing hillsides up to 400 MSL [8] and has been observed in the same regions at least since the year 1771 [10]. Although Icelandic vascular plants originated primarily from Europe, some are known to have originated from the North American continent [11]. However, the origin of the Icelandic strawberry population is uncertain. A comprehensive taxonomic study of the American strawberry genus describes four subspecies of *F*. *vesca* in North America: *F*. *vesca* subsp. *bracteata*, *F*. *vesca* subsp. *vesca*, *F*. *vesca* subsp. *californica*, and *F*. *vesca* subsp. *americana*[4]. However, molecular analysis has suggested that *F*. *vesca* subsp. *bracteata* might be split into two groups based on plastome sequences, which correspond with geography [12]. The proposed geographical distribution of the *F*. *vesca* species and subspecies is shown in Fig 1 [4,13,14]. Hybrids between subspecies could exist in the area where their distribution overlaps, as seen in Fig 1.

Twenty-two wild species are recognized in the *Fragaria* genus [2–4], including the newly discovered *F*. *cascadensis* [15]. In addition, three wild hybrids are known: *F*. × *bifera*, a hybrid of *F*. *vesca* and *F*. *viridis* found in Europe [16], *F*. × *bringhurstii* [17], and *F*. × *ananassa* subsp. *cuneifolia*. The *Fragaria* genus is one of ninety genera in the Rosaceae [18], a family that includes many economically important species such as the octoploid dessert strawberry (*F*. × *ananassa*), apple (*Malus domestica*), pears (*Pyrus* spp.), peach (*Prunus persica*), and roses (*Rosa* spp.) [18], which together make the Rosaceae one of the most economically valuable of all plant families [19].

*F*. *vesca* has repeatedly been proposed as a research model for the Rosaceae [20–22]. Arguments for this include the fact that *F*. *vesca* is a diploid perennial species with a small, fully sequenced genome (240 Mb [20] revised at 206 Mb [18]), an efficient genetic transformation method is available [23], it can be propagated either by seeds or clonally via stolons or branch crowns, and the seed-to-seed cycle is relatively short, only 12–16 weeks [24]. In addition, as a maternal ancestor of *F*. × *ananassa* [3], *F*. *vesca* shares a substantial sequence identity with this economically-important fruit crop. Although, the well-known model plant *Arabidopsis thaliana* does have a smaller genome and is already a favorite in plant research [25], it is usually an annual unlike *F*. *vesca* and it does not suffice for research on perennial-specific traits and development and ripening of fleshy fruit [26]. The wide geographical range of *F*. *vesca* from sub-tropic areas to the arctic and up to 3000 MSL [27] increases its potential as a model for research on adaptive traits. To understand these key traits and their regulation, it is of great importance to analyze natural variability and its selective advantage in certain environments. The value of naturally occurring genetic variation for basic research is already well demonstrated through the use of wild *Arabidopsis* accessions [28, 29].

The use of wild accessions for the study of environmental adaptation requires the comprehensive understanding of the biogeographic patterns of the populations of interest. Large numbers of microsatellite markers have been developed for *Fragaria* species [30–36], with over 4000 SSR markers developed [33] since the sequencing of the *F*. *vesca* genome [20]. These markers facilitate population genomic research in *F*. *vesca*. Furthermore, SSR markers developed in *F*. *vesca* have an observed transferability of over 90% to *F*. × *ananassa* [37], and they have been used to construct linkage maps for *F*. × *ananassa* [33,35].

Strawberries have most likely been consumed by humans for thousands of years [2], but the cultivation of the woodland strawberry is believed to date back only centuries, with the domestication process started by the discovery of a perpetual flowering plant in the low Alps east of Grenoble about 350 years ago [38]. The oldest registered cultivars ‘Rügen’ and ‘Baron Solemacher’, released in 1920 and 1935, respectively [39], are still available along with many others in seed banks and stores. Domestication can greatly affect the distribution of both plants and animals, with domestic varieties known in some cases to return to the wild after human-mediated long-distance dispersal, possibly affecting biogeographic patterns observed through molecular analyses. For research aimed at elucidating biogeography signatures it is therefore important to include samples representing available cultivars.

Reduced diversity in crop plants compared to wild relatives is well recognized in cotton (*Gossypium hirsutum* L.) [40], the potato (*Solanum tuberosum* L.) [41], and the common bean (*Phaseolus vulgaris* L.) [42]. This domestication reduction in diversity is not universally true. For example, apple (*Malus domestica*), a perennial crop plant, has not undergone any significant loss in diversity during the last 800 years [43]. Moreover, maize contains about 60–80% of the diversity of its ancestor teosinte [44, 45]. Also, einkorn wheat, one of the first domesticated crops, has not undergone any considerable diversity reduction [46], and domesticated chili peppers show only ~10% reduction in genetic diversity [47]. To effectively assess the reduction of genetic diversity associated with domestication it is necessary to have a fair estimation of the genetic diversity found in the wild relatives. To achieve this, genetic analyses of a collection of wild accessions are needed.

Due to the loss of genetic diversity in crop species, their wild relatives have long been suggested as a potentially valuable source of novel traits [48]. This has been confirmed on multiple occasions, Maxted and Kell [49] reported 291 studies describing attempts to introgress desired traits into 29 crop species from wild relatives and it has been suggested for strawberries by Liston et al. [2]. The trait of day neutrality was introgressed from *F*. *virginiana* subsp. *glauca* into *F*. × *ananassa* [18, 50] and old cultivars have introgression from *F*. *moschata* and *F*. *chiloensis* genomes in their pedigree [51]. However, in practice, the introgression of traits into a desired cultivar through conventional crossing can be very time consuming–nearly impossible in species of different ploidy such as in the case of *F*. *vesca* and *F*. × *ananassa*–with backcrossing and phenotyping taking years or decades in some plant species. However, methods such as marker assisted selection (MAS) or more recent genomic selection [52], and novel methods of genome editing [53], promise to significantly speed up the use of such natural diversity.

*F*. *vesca* is known to possess traits of interest for resistance to both abiotic and biotic stress [1, 4] as well as fruit aroma [54]. Novel traits from wild *Fragaria* species have been introgressed into cultivars in strawberry breeding programs [1, 55]. Warschefsky et al. [48] proposed that future work in using natural variation for breeding should focus on building a broad collection of wild relatives and sequencing of their genomes. To increase our understanding of the biogeography of *F*. *vesca* with the aim of furthering its use in genetic and genomic research and to shed light on the origin of the Icelandic *F*. *vesca* population we undertook a population genomic analysis of 295 *F*. *vesca* samples originating from Eurasia and America, using 56 SSR markers.

## Materials and methods

### A global *Fragaria vesca* collection

Plants or berries were collected from a total of 274 locations in 31 countries and 16 states (US) around the world (S1 Table) with the aim of creating a global collection representing the current wild distribution of *F*. *vesca*. Despite our best efforts we were not able to fully cover the current global distribution of introduced *F*. *vesca*, with samples missing from areas such as Hawaii, New Zealand, Australia, southern Africa, Madagascar, the Canary Islands, and the Cape Verde Islands, as well as several South American countries. Additionally, 26 cultivars were included in the study, giving a total number of 298 plants. In total, 232 Eurasian plants were analyzed (not including cultivars and outgroups), including 54 from Iceland, 37 accessions originating from North America, one from South America, and two from Japan. Also, two species other than *F*. *vesca* were included as outgroups: *F*. *chinensis* from China and *F*. *viridis* from Sweden (both accessions came taxonomically identified from USDA Germplasm Resources Information Network—GRIN). All sampling was done in accordance with regional laws and regulations governing the collection of plant material for research purposes. Accession numbers for material received from GRIN are listed in S1 Table. The distribution of sampling sites for all wild samples is shown in Fig 1.

### DNA isolation, marker amplification and fragment detection

Genomic DNA was extracted from homogenized young leaf tissue using the DNeasy 96 Plant Kit or DNeasy Plant Mini Kit from QIAGEN^®^ (Valencia, CA). The DNeasy 96 Plant Kit protocol was modified for use with the available equipment. a Universal 320 centrifuge (Hettich GmbH & Co.) with the maximum of 4000 RPM in a Hettich 1460 rotor. The amount of DNA extracted with the DNeasy 96 Plant Kit protocol was measured using NanoDrop 1000 (Thermo Scientific).

Samples of 300 individuals (S1 Table) were analyzed using 68 microsatellite markers (S2 Table) [33, 56–58]. All markers used here are expressed simple sequence repeats (EST-SSR) markers, which are, although reported to be less polymorphic than non-genic SSR markers, of great value for population structure analysis due to their transferability between species (due to the higher conservation of genic sequences) and the fact that they make up for the lower levels of polymorphism, compared to non-genic markers, by being concentrated in gene-rich regions [59]. The microsatellite markers were amplified using a TProfessional 384 thermocycler (www.biometra.de) with a 5 μl reaction volume containing 0.6 ng of genomic DNA in 1X PCR buffer (Bioline, London, UK), 3 mM MgCl2, 0.08U of BIOTAQ DNA polymerase (Bioline), 0.8 mM dNTPs, and 0.4 μM of each primer. A modified touchdown PCR protocol was followed, as described by Sato et al. [60]. The PCR products were separated by an ABI 3730xl fluorescent fragment analyzer (Applied Biosystems). The polymorphisms were investigated using GeneMarker software (http://softgenetics.com/).

### Analysis of genetic diversity and population structure

Descriptive statistics were calculated using GenAlEx 6.501 [61, 62] for each microsatellite marker, including the number of observations (N) for each marker, number of alleles (N_a_) per locus, and both observed (H_o_), and expected (H_e_) heterozygosity. For a population-wide analysis GenAlEx 6.501 was used to calculate the average number of alleles per population (N_ap_), number of effective alleles (N_e_), number of private alleles (N_PA_), observed (Ho) and expected (He) heterozygosity, and the fixation index (F = 1-(*H*_*o*_/*H*_*e*_)). GenAlex was also used for principal coordinate analysis (PCoA) and pairwise population F_st_ values (F_ST_ = 1- (average H_e_/H_T_)). Presence of null alleles was tested using Freena [63]. Additional statistics were calculated in PowerMarker 3.25 [64], including the polymorphic information content (PIC) and the major allele frequencies (MAF) for each marker. PowerMarker was also used to construct an evolutionary distance matrix based on Nei et al.’s *D*_*A*_ distance method [65]. A phylogenetic tree of a split network based on this matrix was drawn up using SplitsTree 4 [66]. Mega version 5 [67] was used to reconstruct a phylogenetic tree using the neighbor-joining method [68] with bootstrap values.

To identify the number of populations and admixtures, the dataset was analyzed using the admixture model of Structure 2.3.4 [69–72] and the Markov Chain Monte Carlo (MCMC) method for estimation of probabilities. All loci were assumed to be independent and in linkage equilibrium. Populations were not pre-described. All Structure runs were repeated 5 times for each K from 1–20 for the whole dataset and for each *K* from 1–10 for the ‘American’ and ‘Eurasian’ data-sets. The MCMC method was run with a burn-in period of 50,000 and 10,000 repetitions. Other settings were by default. Structure Harvester [73] was used to find the optimal number of clusters (*K*) for each dataset, where the average likelihood values K (L(*K*)) for each run were used to find Δ*K*, i.e., the rate of change in *ln*Pr(*X*|*K*), since the maximum value of L(*K*) can give an overestimate of clusters [74].

## Results

### Descriptive statistics of microsatellite markers

Of the 68 markers amplified, 10 were monomorphic and therefore uninformative. In addition, more than two alleles were repeatedly observed per sample for markers *FVES2533* and *FVES0634*. As this is not consistent with the diploid nature of *F*. *vesca*, these markers were excluded from further analysis. Descriptive statistics for each of the remaining 56 markers used are listed in Table 1. The mean number of observed individuals per marker (*N*) was 289.9. The 56 polymorphic markers had numbers of alleles ranging from 2–16 for all samples, with a total of thirteen bi-allelic markers. A total of 250 alleles was observed for the 56 markers, giving a mean number of 4.46 alleles per locus. The observed heterozygosity (*H*_*o*_) ranged from zero for thirteen markers to 0.938 for marker *FVES3440* with a mean *H*_*o*_ of 0.075. The expected heterozygosity (*H*_*e*_) ranged from 0.003 for marker *FAES0293* to 0.797 for marker *FVES0109*, with a mean of 0.170. The polymorphic information content (PIC) ranged from 0.003 for marker *FAES0293* to 0.78 for marker *FVES0109*, with a mean of 0.151. The major allele frequency (MAF) ranged from 0.393 for marker *FVES0109* to 0.998 for marker *FAES0293*, with an average of 0.885.

**Table 1:** Summary statistics of markers tested.

Marker	N	N_a_	H_o_	H_e_	PIC	Null_a_	MAF	Marker	N	N_a_	H_o_	H_e_	PIC	Null_a_	MAF
FAES0093	295	4	0.034	0.085	0.084	0.60	0.956	FVES1201	294	3	0.003	0.007	0.007	0.50	0.997
FAES0208	294	4	0.010	0.069	0.068	0.85	0.964	FVES1213	294	7	0.065	0.455	0.410	0.86	0.707
FAES0293	295	2	0.003	0.003	0.003	0.00	0.998	FVES1230	295	2	0.000	0.007	0.007	1.00	0.997
FAES0357	291	2	0.000	0.163	0.149	1.00	0.911	FVES1313	289	5	0.017	0.406	0.372	0.96	0.751
FAES0376	294	2	0.000	0.007	0.007	1.00	0.997	FVES1356	295	2	0.000	0.007	0.007	1.00	0.997
FAES0465	288	5	0.000	0.028	0.027	1.00	0.986	FVES1362G	295	4	0.000	0.020	0.020	1.00	0.990
FAES0479	276	2	0.011	0.011	0.011	0.00	0.995	FVES1392	294	2	0.014	0.014	0.013	-0.01	0.993
FP0380	292	2	0.000	0.007	0.007	1.00	0.997	FVES1470	281	4	0.007	0.042	0.041	0.83	0.979
FP0488	260	8	0.019	0.122	0.119	0.84	0.937	FVES1621	295	2	0.000	0.007	0.007	1.00	0.997
FVES0109	291	16	0.206	0.797	0.780	0.74	0.393	FVES1640	295	5	0.051	0.297	0.257	0.83	0.820
FVES0128	294	9	0.109	0.504	0.422	0.78	0.624	FVES1711	295	2	0.000	0.007	0.007	1.00	0.997
FVES0233	293	5	0.020	0.207	0.188	0.90	0.884	FVES1724	293	7	0.089	0.361	0.314	0.75	0.775
FVES0381	285	7	0.032	0.095	0.093	0.67	0.951	FVES1793	294	2	0.000	0.007	0.007	1.00	0.997
FVES0392	295	4	0.075	0.423	0.348	0.82	0.708	FVES1816	295	3	0.125	0.124	0.119	-0.01	0.934
FVES0435	295	6	0.088	0.404	0.338	0.78	0.731	FVES1877	295	4	0.007	0.010	0.010	0.33	0.995
FVES0459	293	7	0.010	0.060	0.060	0.83	0.969	FVES1907	295	3	0.003	0.010	0.010	0.67	0.995
FVES0463	293	3	0.024	0.101	0.098	0.76	0.947	FVES2235	288	3	0.049	0.285	0.246	0.83	0.828
FVES0480	235	8	0.302	0.391	0.361	0.23	0.764	FVES2316	295	3	0.003	0.017	0.017	0.80	0.992
FVES0513	294	3	0.003	0.037	0.036	0.91	0.981	FVES2349	294	4	0.007	0.020	0.020	0.66	0.990
FVES0567	294	5	0.010	0.047	0.046	0.78	0.976	FVES2369	295	6	0.037	0.206	0.190	0.82	0.885
FVES0577	295	3	0.000	0.378	0.309	1.00	0.749	FVES2661	294	5	0.017	0.027	0.027	0.37	0.986
FVES0794	294	5	0.085	0.562	0.465	0.85	0.485	FVES2882	295	2	0.000	0.007	0.007	1.00	0.997
FVES0960	294	6	0.007	0.180	0.157	0.96	0.912	FVES2901	293	2	0.000	0.007	0.007	1.00	0.997
FVES0989	295	4	0.017	0.037	0.037	0.54	0.981	FVES3274	292	5	0.007	0.073	0.072	0.91	0.962
FVES1031	294	6	0.003	0.034	0.034	0.90	0.983	FVES3330	295	2	0.112	0.106	0.100	-0.06	0.944
FVES1070	294	4	0.003	0.024	0.024	0.86	0.988	FVES3346	271	5	0.697	0.491	0.397	-0.42	0.622
FVES1156	295	4	0.017	0.088	0.086	0.81	0.954	FVES3440	288	3	0.938	0.504	0.382	-0.86	0.517
FVES1160	242	7	0.446	0.478	0.418	0.07	0.676	FVES3693	288	10	0.417	0.662	0.625	0.37	0.523
								**Average**	**289.9**	**4.46**	**0.075**	**0.170**	**0.151**	**0.11**	**0.885**

N, number of individuals analyzed for each marker; N_a_, average number of alleles for each marker; H_o_, observed heterozygosity; H_e_, expected heterozygosity; PIC, polymorphism information content; Null_a_, presence of null alleles; MAF, major allele frequency.

Of the 298 accessions in the collection, three samples, one *F*. *vesca subsp*. *americana* (ID 28) and two Eurasian *F*. *vesca subsp*. *vesca* (ID numbers 145 and 146), were excluded from the analysis due to a higher than expected number of alleles per marker. In these samples, the average number of alleles per locus was 2.4, 2.4 and 2.5, respectively, indicating that they might be polyploids or the results of mixed samples. In addition two Icelandic samples were omitted from analysis due to a labelling mistake.

### Population structure and genetic diversity

Descriptive statistics for each proposed population are listed in Table 2. The highest mean number of alleles, 1.98, was found in the Eurasian group (excluding Iceland) and the lowest in the Japanese samples 1.08. *F*. *vesca* subsp. *vesca* showed the highest values for *N*_*e*_ = 1.26, *H*_*o*_ = 0.11 and *H*_*e*_ = 0.15. The highest frequency of private alleles, N_PA_ = 0.30, was found in the *F*. *vesca* subsp. *bracteata* ‘Rocky Mts’ group, with the ‘Pacific Coast’ group tied at 0.26 with the Eurasian group.

**Table 2:** Results of microsatellite analysis by populations.

	N	N_ap_	N_e_	N_PA_	H_o_	H_e_	F
*F*. *vesca* subsp. *americana*	5	1.29±0.07	1.18±0.06	0.11±0.04	0.06±0.02	0.10±0.02	0.41±0.08
*F*. *vesca* subsp. *bracteata* ‘Rocky Mts’	7	1.76±0.12	1.39±0.07	0.30±0.08	0.09±0.02	0.19±0.03	0.49±0.06
*F*. *vesca* subsp. *bracteata* ‘Pacific Coast’	13	1.53±0.11	1.22±0.05	0.26±0.07	0.09±0.02	0.12±0.02	0.26±0.06
*F*. *vesca* subsp. *californica*	4	1.20±0.06	1.15±0.05	0.03±0.02	0.08±0.03	0.09±0.02	0.05±0.10
Cultivars	26	1.48±0.12	1.14±0.04	0.03±0.02	0.04±0.02	0.08±0.02	0.59±0.07
Eurasian *F*. *vesca* subsp. *vesca*	176	1.98±0.21	1.23±0.07	0.26±0.08	0.06±0.02	0.11±0.02	0.42±0.06
Icelandic *F*. *vesca* subsp. *vesca*	52	1.52±0.11	1.14±0.05	0.06±0.03	0.05±0.02	0.07±0.02	0.41±0.08
Japanese *F*. *vesca* subsp. *vesca*	2	1.08±0.04	1.08±0.04	0.00±0.00	0.05±0.03	0.05±0.02	0.00±0.13
American *F*. *vesca* subsp. *vesca*	8	1.62 ±0.12	1.26±0.05	0.02±0.02	0.11±0.03	0.15±0.02	0.22±0.07
Total		1.41±0.03	1.16±0.02		0.09±0.01	0.10±0.01	0.23±0.03

N, number of individuals in each population; N_a_, average number of alleles over all markers; N_e_, number of effective alleles; N_PA_, number of private alleles unique to a single population; H_o_, observed heterozygosity; H_e_, expected heterozygosity; F, fixation index. Standard error (±SE) is shown for all averages.

**Fig 2:**
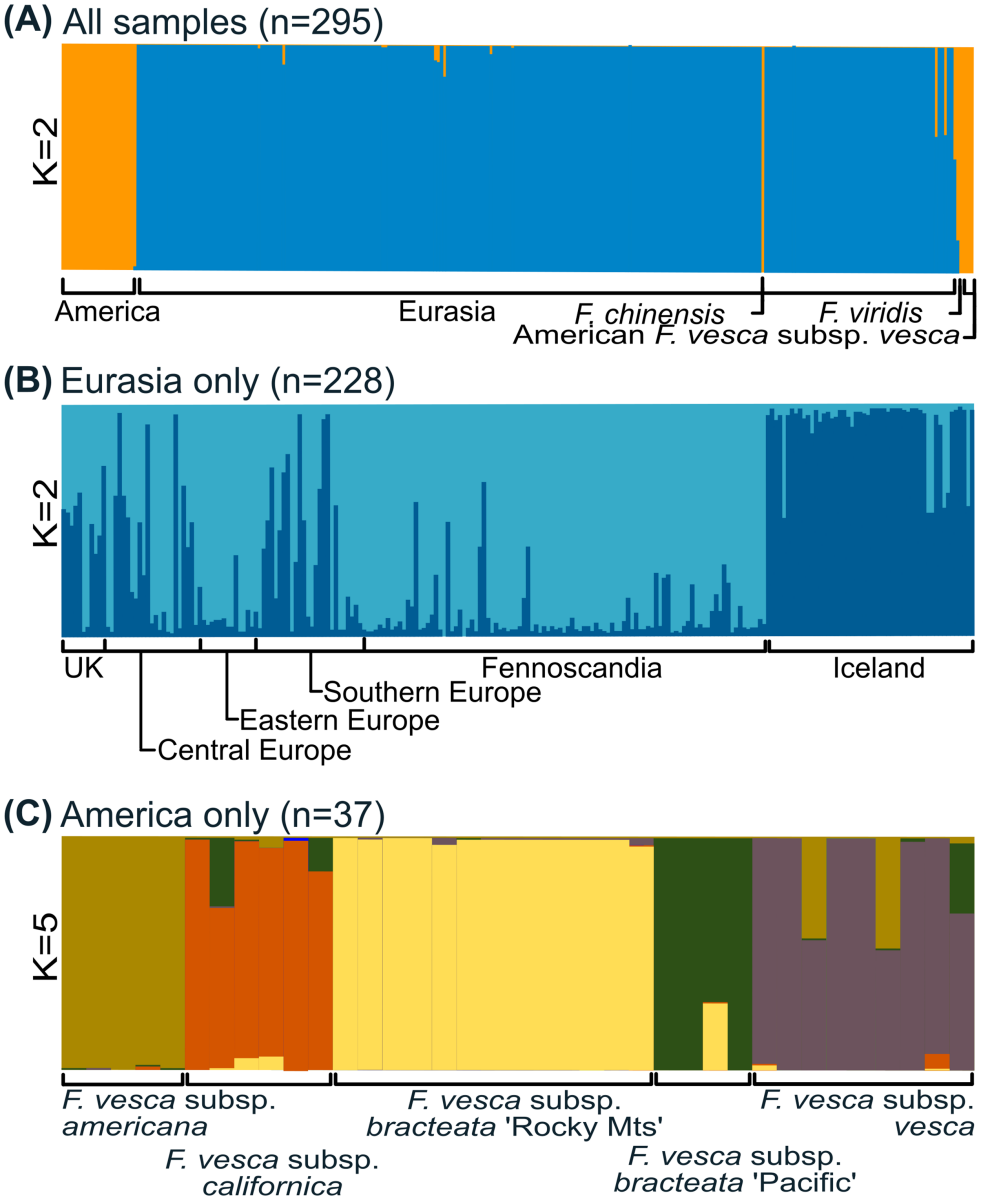
Results of Structure analyses. (A) Analysis of the whole data set including cultivars. (B) Eurasian samples without the cultivars. (C) American samples only. The labels show the origin of samples based on populations proposed by (Hilmarsson, 2015).

**Fig 3:**
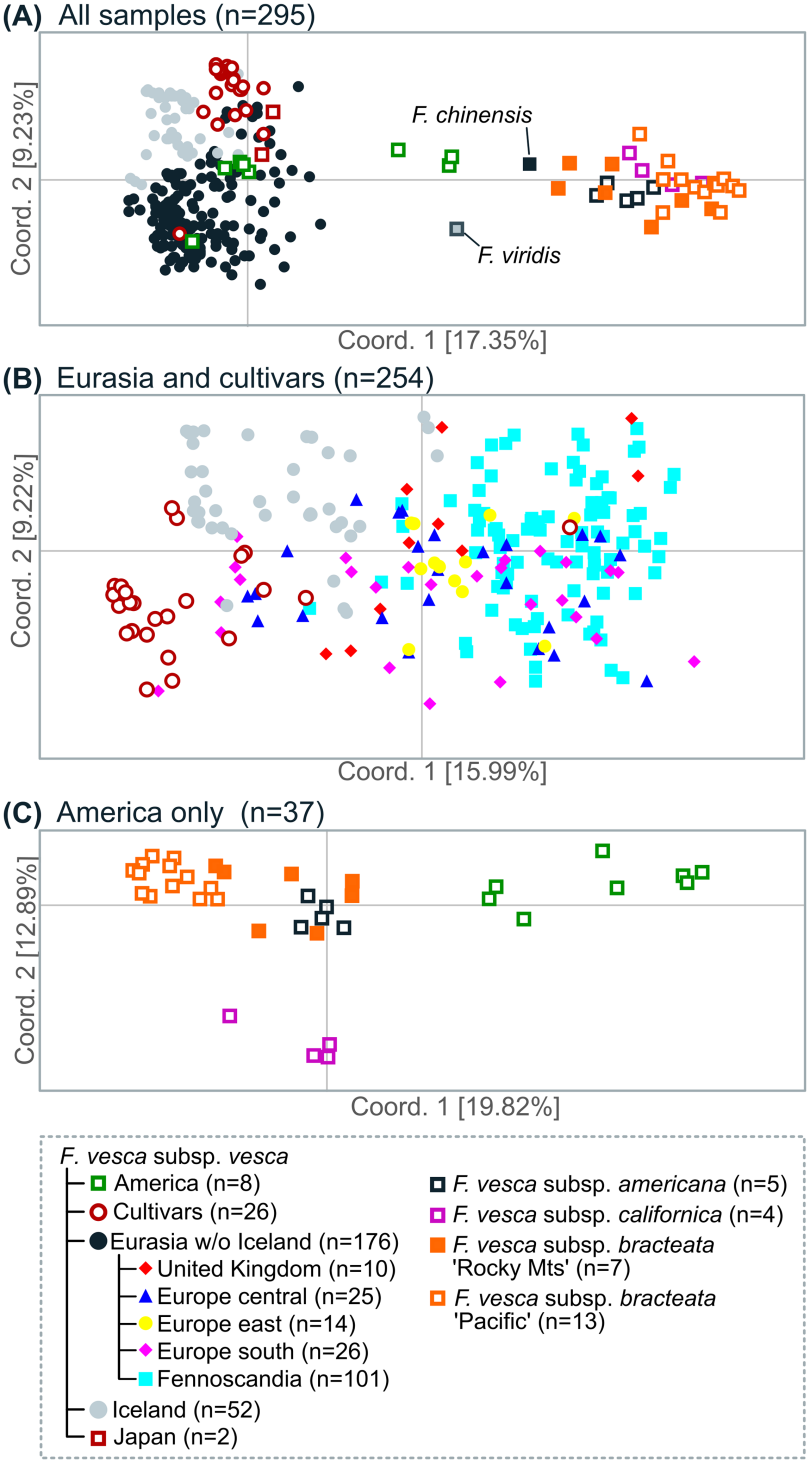
Principal coordinate analysis of *F*. *vesca* microsatellite data. (A) Analysis of the whole data set, including cultivars. (B) Eurasian samples with cultivars. (C) American samples only. The clusters suggested based on the Structure analysis are color coded.

The Structure admixture results for the whole dataset, including all wild individuals and cultivars (*n* = 295), suggest that the collection should be split into two sub-populations, with K = 2 (Δ*K* = 127.56) (S1 Fig and Fig 2A). This analysis groups cultivars with the Eurasian samples, while clearly separating the Eurasian and American samples. This is somewhat corroborated by the PCoA of all individuals, which shows a strong separation of the Eurasian and the American samples (Fig 3A), while the American *F*. *vesca* subsp. *vesca* samples are either mixed with the Eurasian samples or end up between the two groups (Fig 3A). Another PCoA shows the cultivars overlapping with the central European samples (Fig 3B). Structure analysis with all Eurasian samples including cultivars resulted in two clusters (*K* = 2), one containing all wild samples and the other containing all cultivars, which is in line with the results of the PCoA (Fig 3A). To further test for divergence within the Eurasian group, the analysis was performed without the cultivars. This analysis on 228 Eurasian samples suggested the presence of two clusters (*K* = 2; Δ*K* = 28.13) (S1 Fig and Fig 2B), separating the Icelandic samples from the rest. Again, the PCoA for the Eurasian samples (Fig 3B), which explains a total of 25.21% of the variability on the first two axes, gives support to the Structure results, showing that the Icelandic samples separate from the other Eurasian samples and are most divergent from the Fennoscandian group, with some overlap with the cultivars and samples originating mostly from central Europe and the UK, as can be seen on a phylogenetic tree between all individuals (S2 Fig).

Structure analysis of the American samples (*n* = 37) suggests that five clusters (*K* = 5) is the appropriate number (Δ*K* = 16.61) (S2 Fig) for the wild American samples (Fig 2C), with three clusters consisting of previously identified subspecies and *F*. *vesca* subsp. *bracteata* split into two clusters referred to here as ‘Pacific Coast’ and ‘Rocky Mts’ groups based on their geographical origin (Fig 2C). The PCoA analysis for the American samples (Fig 3C) explained a total of 32.71% of the variability on the first two axes and does lend some support to the Structure results.

**Fig 4:**
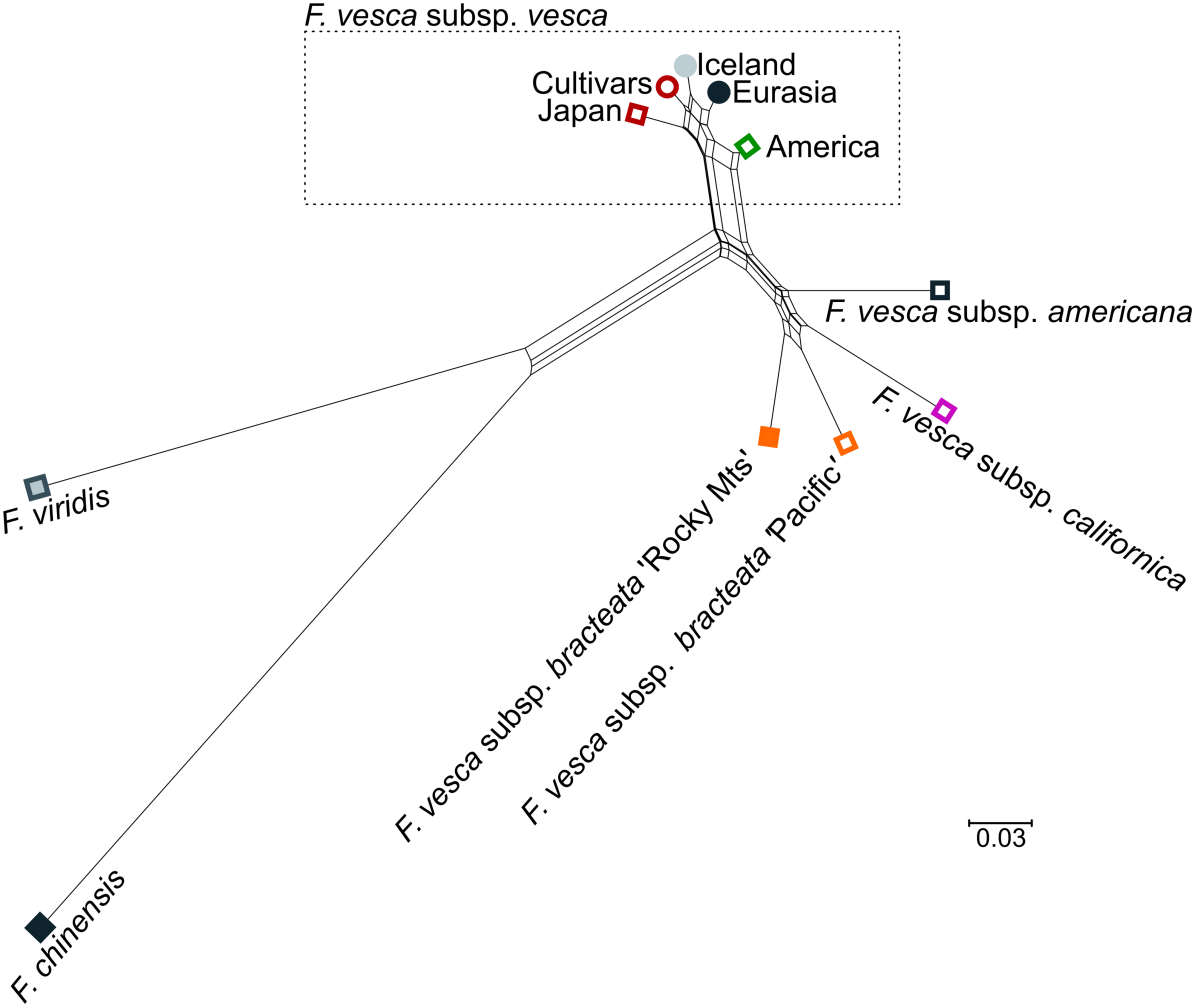
A SplitsTree analysis based on genetic distance between groups. Groups are color coded as in Fig 3.

Nei et al.’s [65] *D*_*A*_ distance was calculated for the groups presented here (S3 Table) and the results presented in a network diagram (Fig 4). The results show a clear separation between much of the American and the Eurasian samples, except for the American *F*. *vesca* subsp. *vesca* samples which group close to the Eurasian samples.

## Discussion

In the study presented here a total of 68 EST-SSR markers, of which were 56 polymorphic, were used to analyze a global collection of 295 *F*. *vesca* samples from 274 locations in 31 countries and 16 states (US). The diversity observed for each of the markers was relatively low, with a mean number of alleles of only 4.5. These values were much lower than seen in some recent studies on Rosaceae species such as the mean number of alleles of 18.7 observed in the almond (*Prunus dulcis* (Mill.)) [75] and 10.8 in *F*. × *ananassa* [51], yet similar to other results observed in *F*. *vesca*, where 4.9 was the mean number of alleles from 21 microsatellites in fifteen *F*. *vesca* samples [32]. The question arises whether this reflects a poor choice of microsatellites or whether this rather reflects low levels of genetic diversity in the populations under study. The relative uniformity of alleles for each of the markers analyzed and the results of Hadonou et al. [32] might suggest that the values seen here reflect low levels of genetic diversity in the species, but it can be pointed out that although the average number of alleles was low, the most polymorphic marker revealed 16 alleles. The mean frequencies of null alleles for all the markers was 0.11. This could have been due to DNA quality leading to genotyping error, which would also explain the nine markers that revealed two alleles but high major allele frequencies. The selfing nature of *F*. *vesca* could also lead to an overestimate of null allele frequencies.

The mean observed heterozygosity (*H*_*o*_) found here was 0.075 for the whole collection, considerably lower than the average expected heterozygosity (*H*_*e*_) of 0.170. These values are then much lower than values seen, for example, in *Prunus sibirica*, a highly outcrossing species, with values of *H*_*o*_ = 0.639 and *H*_*e*_ = 0.774 [76]. One likely explanation for the discrepancy seen between *H*_*o*_ and *H*_*e*_ might be the existence of subpopulations within the global collection, that is the North American subspecies, confirmed here through various means, such as Structure analyses. When values for *H*_*o*_ and *H*_*e*_ were compared for individual subpopulations suggested here the observed heterozygosity was always lower than the expected heterozygosity, except for the Japanese samples where they were equal (Table 2). The large difference within the *F*. *vesca* subsp. *bracteata* ‘Rocky Mts’ group (*H*_*o*_ = 0.09, *H*_*e*_ = 0.19) is surprising considering the gynodioecy within the group [12]. *F*. *vesca* is a self-compatible species and low levels of observed heterozygosity have previously been reported [32, 77]. Low levels of heterozygosity could indicate low cross-fertilization and high selfing rates, but might also be explained by the asexual dispersal by means of stolons [12] or a Wahlund effect, as observed in the Siberian apricot [76]. The low H_e_ seen here, especially in some groups, e.g. the Icelandic samples, could be the result of a recent bottleneck since expansion leads to a reduced diversity [78] (Table 2). The cultivar group exhibited low diversity with the highest fixation index of all groups F = 0.59 (Table 2). The PCoA showed the tight cluster of the individuals analyzed and its divergence from the wild samples (S2 Fig). The Icelandic samples analyzed here were most closely related to the cultivars (S3 Table and visualized in Figs 3B and 4 and S2 Fig), but overlapped also with central and southern European samples.

The principal coordinate analyses performed here revealed a great difference between the Eurasian (without Iceland) and American (without American *F*. *vesca* subsp. *vesca*) groups (Fig 3) with genetic distances from 0.170–0.204 and *F*_st_ values from 0.194–0.354 (S3 Table). In addition, the Structure analysis placed American and Eurasian samples into separate clusters and a detailed analysis of American samples showed five clusters which consisted of the four subspecies identified by Staudt [4] (Fig 2). The morphological diagnosis by Staudt did not fully reveal this large difference between the endemic American subspecies and the subsp. *vesca*. However, these results could complement the results of Njuguna [12] where subsp. *bracteata* did split into two groups divided by the Great Basin in the western US, much as presented here, possibly because of genetic variation in loci determining sexual phenotypes [79], but differences in cytoplasmic haplotypes have been reported, with western populations dominated by one chlorotype and populations from the Rocky Mts by another [12, 79]. No evidence of hybrids between the subsp. *bracteata* and subsp. *americana* was revealed in the admixture analyses as suggested by Staudt [4] and reported by Stanley et al. [79], but this is most likely best explained by the limited sampling of the two subspecies in the current study. However, there seem to be two hybrids between subsp. *americana* and subsp. *vesca* and one between subsp. *bracteata* and subsp. *vesca*, as seen in both the admixture analyses (Fig 2C) and the PCoA analyses (Fig 3A and 3C), and in all three clusters with subsp. *vesca*. Hybrids between subsp. *americana* and subsp. *vesca* can be natural since their area of distribution in the northeastern United States overlap, but natural populations of subsp. *bracteata* are not known in this region (Fig 1), although they could have been introduced as suggested by Stanley [79]. The samples that were collected in America that group together with the European samples are categorized as *Fragaria vesca* subsp. *vesca* (Fig 1). These samples were collected in the northeastern United States, where cultivars were already being grown at the beginning of the last century [38] and therefore most likely introduced. The same conclusion can be made for the Japanese samples, as already suggested by Hultén [13], and the single Bolivian sample included in the study. The American samples all came from the GRIN germplasm and they could represent greater levels of diversity on average than observed in nature; since what gets collected and curated might be skewed in favor of phenotypically unusual individuals, leading to greater genetic diversity, as noted by Chambers et al. [80]. The number of individuals and markers affect the detection of clusters in Structure [74]. Some of the sample groups analyzed here were very small; for example, the outgroups only contained single representatives of proposed populations, samples from Japan and the *F*. *vesca* subsp. *californica* contained only two and four samples, respectively. It should also be mentioned that because the Evanno method calculates the mean difference between the successive likelihood values of *K*, there is no ΔK value for *K* = 1.

It is also important to mention that to maximize the accuracy of genetic distance calculations, the number of samples need to be 100 or more, although this also depends on the polymorphism of the markers used [81]. In many cases the sample collection analyzed here did not fulfill this requirement, and further studies, with larger samples and more markers or even whole genome sequencing, are therefore recommended.

It has been demonstrated that the admixture model implemented by Structure can detect the most likely number of clusters even if the samples contain low genetic variation [71]. In the Eurasian samples, where genetic variation was low, Structure revealed two clusters, suggesting two genetic populations among the genotypes collected in Eurasia (Fig 2B), with the grouping of the Eurasian samples consistent in all analytical methods used (Figs 2, 3 and 4). Despite this it is important to note that in the PCoA of individual samples there was a clear overlap between the two clusters, the Icelandic samples and those from the rest of Eurasia. Based on our results, the origin of the Icelandic strawberry population was clearly Eurasian and not American, but interestingly our analysis did not group the Icelandic samples with the Fennoscandian samples but rather showed more genetic similarity with cultivars and central European samples. This close relationship is also seen in the overlap of the two groups in the PCoA (Fig 3B). A phylogenetic tree of individuals branches the Icelandic group off from the rest of the Eurasian samples and shares a branch with most of the cultivars and some central and southern European samples (S2 Fig). One possible explanation for these results might be that the Icelandic strawberries represent a population descended from the same stock that gave rise to the modern *F*. *vesca* cultivars. However, they cannot have been recently introduced since they have been growing in the same locations for at least 250 years [10]. The possible presence of *F*. *viridis* (as *F*. *collina*) in Iceland has been reported [82] but has not been conclusively demonstrated and we found no evidence of *F*. *viridis* or of *F*. × *bifera* in this study.

The use of populations of crop wild relatives as research models has been suggested as an approach to disseminating genetic pathways of importance to adaptation [29]. For such an approach, a collection of wild material is of great importance, and bearing that in mind, we gathered a global collection of *F*. *vesca* plants and compared them with cultivars of the same species. Through our initial analysis of biogeography and genetic diversity within this worldwide collection we have confirmed the previous classification of *F*. *vesca* into subspecies using molecular markers, and we have shown that the cultivars chosen are homogeneous and group together with the Eurasian samples. Our data also divide European subsp. *vesca* into two groups, one consisting of an Icelandic group and some accessions from southern and central Europe, and another consisting of the rest of the Europe, although not without overlap between groups. The clear divergence between the Icelandic group and the Fennoscandian does not correlate with results for other floral species in Iceland which are related to Nordic groups [11]. We find no evidence for any population sub-structuring within the Icelandic population despite sourcing material from around the country. Further studies with more markers and possibly with a larger number of samples or samples focusing on certain geographical areas are needed to define more detailed biogeographical patterns of *F*. *vesca*.

## Supporting information

S1 Fig Results of ΔK from the structure Harvester. (A) Whole data set, including cultivars. (B) Eurasian samples without cultivars. (C) American samples only.
(TIF)

S2 Fig A neighbor-joining tree showing the genetic distance between all individuals tested. The tree is rooted to the two other species used in the study, *F*. *chinensis* and *F*. *viridis*. Information about bootstrap values above 50 that are not at the end of branches.
(TIF)

S1 Table Information on sampling locations. (DOCX)

S2 Table Information on microsatellite markers used. Including marker name, sequence of forward and reverse primers, GenBank accession number (Acc. no.), pattern of repeats (Repeat), chromosome location (Chr.), the linkage position (Linkage), and species name.
(DOCX)

S3 Table Nei’s genetic distance and pairwise population Fst values. Nei’s genetic distance (below diagonal line) and pairwise population Fst values (above diagonal line) between groups.
(DOCX)
