# Supplementary figures and images for: Population genetic analysis of a global collection of Fragaria vesca using microsatellite markers
Source: PLoS One. 2017 Aug 30;12(8):e0183384. doi: 10.1371/journal.pone.0183384 (PMC5576660; doi:10.1371/journal.pone.0183384)

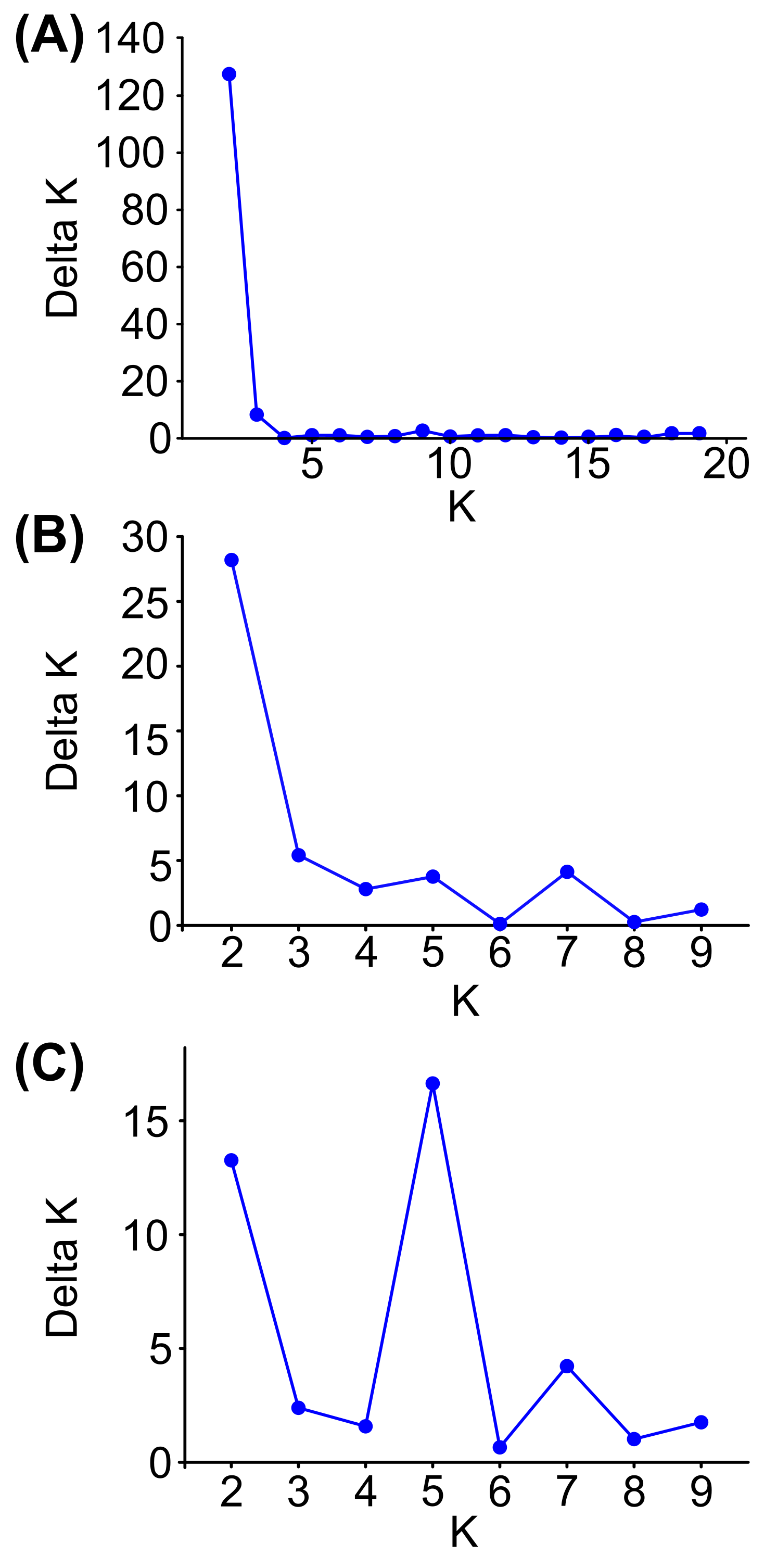

Supplement: S1 Fig — (A) Whole data set, including cultivars. (B) Eurasian samples without cultivars. (C) American samples only. (TIF) [file pone.0183384.s001.tif]

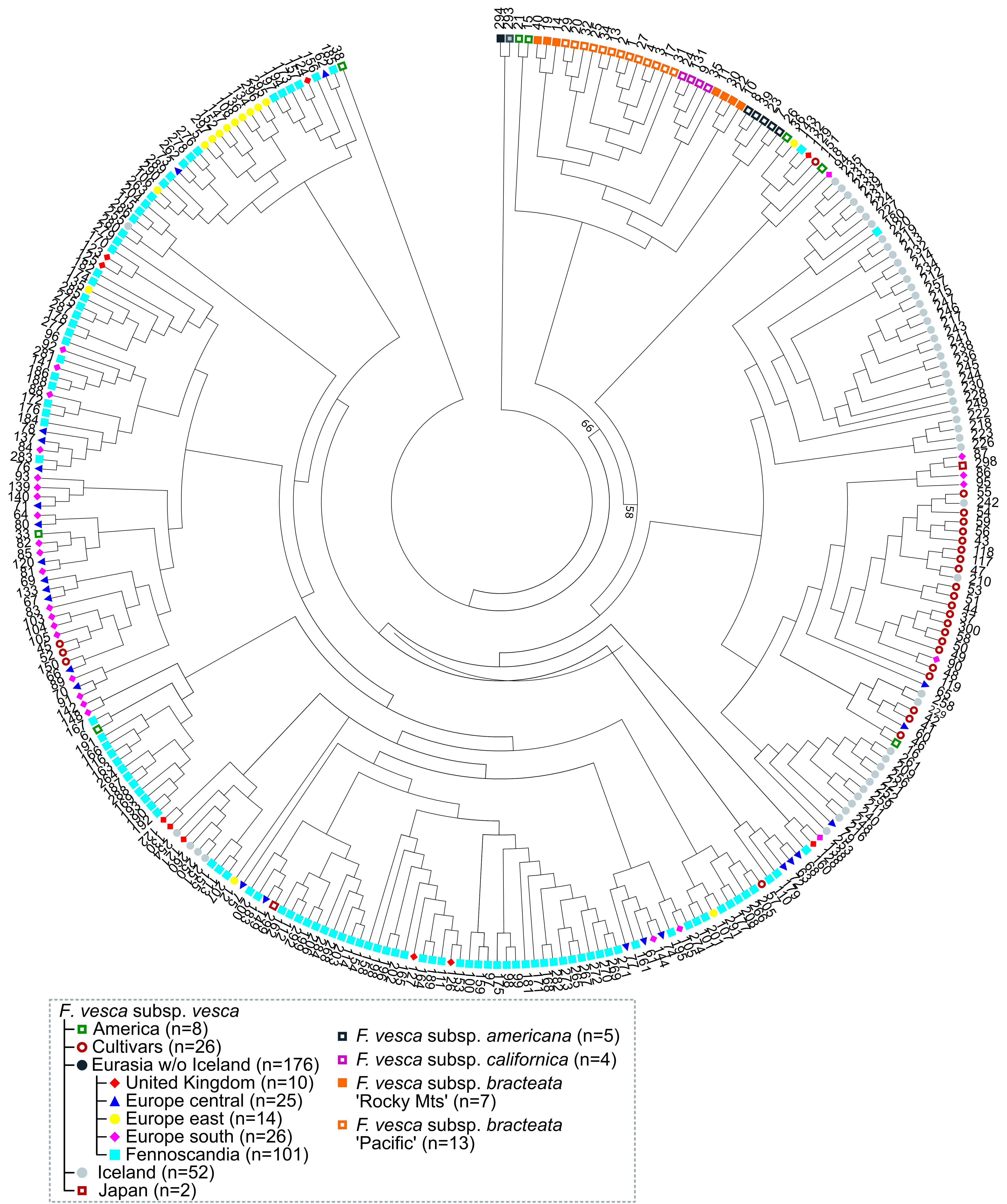

Supplement: S2 Fig — The tree is rooted to the two other species used in the study, F. chinensis and F. viridis. Information about bootstrap values above 50 that are not at the end of branches. (TIF) [file pone.0183384.s002.tif]
