# Supplementary material for: Population genetic analysis of a global collection of Fragaria vesca using microsatellite markers
Source: PLoS One. 2017 Aug 30;12(8):e0183384. doi: 10.1371/journal.pone.0183384 (PMC5576660; doi:10.1371/journal.pone.0183384)
Supplement: S1 Table — (DOCX) [file pone.0183384.s003.docx]

**S1 Table. Information on sampling locations.**

| **ID** | **Group** | **Country** | **Species** | **Coordinates** | | **Material** | | **Collector/Supplier** | |
| --- | --- | --- | --- | --- | --- | --- | --- | --- | --- |
| 47 | Cultivar | Seed store | 'Ali Baba' | -; - | | Seed | | Strawberryseedstore.com | |
| 49 | Cultivar | Seed store | 'Aromaatne Üllatus' | -; - | | Seed | | Strawberryseedstore.com | |
| 50 | Cultivar | Seed store | 'Belaja Dusha' | -; - | | Seed | | Strawberryseedstore.com | |
| 59 | Cultivar | Seed store | 'di Bosco' | -; - | | Seed | | Strawberryseedstore.com | |
| 55 | Cultivar | Seed store | 'Fragola Quattri Stagioni' | -; - | | Seed | | Strawberryseedstore.com | |
| 43 | Cultivar | Seed store | 'Mignonette' | -; - | | Seed | | Strawberryseedstore.com | |
| 53 | Cultivar | Seed store | 'Red Wonder' | -; - | | Seed (A) | | Strawberryseedstore.com | |
| 54 | Cultivar | Seed store | 'Red Wonder' | -; - | | Seed (B) | | Strawberryseedstore.com | |
| 46 | Cultivar | Seed store | 'Regina' | -; - | | Seed | | Strawberryseedstore.com | |
| 44 | Cultivar | Seed store | 'Reines des Vallees' | -; - | | Seed | | Strawberryseedstore.com | |
| 45 | Cultivar | Seed store | 'Rodluvan' | -; - | | Seed (A) | | Strawberryseedstore.com | |
| 52 | Cultivar | Seed store | 'Rodluvan' | -; - | | Seed (B) | | Strawberryseedstore.com | |
| 42 | Cultivar | Seed store | 'Ruegen' | -; - | | Seed (A) | | Strawberryseedstore.com | |
| 56 | Cultivar | Seed store | 'Ruegen' | -; - | | Seed (A) | | Strawberryseedstore.com | |
| 57 | Cultivar | Seed store | 'Runnerless' | -; - | | Seed | | Strawberryseedstore.com | |
| 51 | Cultivar | Seed store | 'Surprise of September' | -; - | | Seed | | Strawberryseedstore.com | |
| 58 | Cultivar | Seed store | 'White Solemacher' | -; - | | Seed | | Strawberryseedstore.com | |
| 41 | Cultivar | Seed store | 'Yellow Cream' | -; - | | Seed | | Strawberryseedstore.com | |
| 48 | Cultivar | Seed store | 'Zolotoi Desert' | -; - | | Seed | | Strawberryseedstore.com | |
| 294 | Eurasia/outgroup | China | *F*. *chinensis* | 31.50991; 110.33281 | | Plant | | USDA GRIN (PI551576) | |
| 5 | Eurasia | Russia | *F. vesca* ssp. *vesca* | 54.910418; 82.902026 | | Plant | | USDA GRIN (PI552240) | |
| 12 | Eurasia | Russia | *F. vesca* ssp. *vesca* | 54.910418; 82.902026 | | Plant | | USDA GRIN (PI552239) | |
| 60 | Eurasia | Austria | *F. vesca* ssp. *vesca* | 47.25; 13.16667 | | Plant | | Sigridur Dalmannsdottir | |
| 61 | Eurasia | Czech | *F. vesca* ssp. *vesca* | 50.795996; 14.107753 | | Plant | | Timo Hytonen | |
| 62 | Eurasia | Denmark | *F. vesca* ssp. *vesca* | 55.567359; 9.731359 | | Plant | | Timo Hytonen | |
| 63 | Eurasia | France | *F. vesca* ssp. *vesca* | 55.215089; 11.463444 | | Plant | | Timo Hytonen | |
| 64 | Eurasia | France | *F. vesca* ssp. *vesca* | 41.430376; 9.172636 | | Plant | | Timo Hytonen | |
| 65 | Eurasia | France | *F. vesca* ssp. *vesca* | 48.804324; 2.116268 | | Plant | | Timo Hytonen | |
| 66 | Eurasia | France | *F. vesca* ssp. *vesca* | 50.015208; 2.6978 | | Plant | | Timo Hytonen | |
| 67 | Eurasia | Germany | *F. vesca* ssp. *vesca* | 48.421779; 7.719479 | | Plant | | Timo Hytonen | |
| 68 | Eurasia | Germany | *F. vesca* ssp. *vesca* | 51.823766; 11.295645 | | Plant | | Timo Hytonen | |
| 69 | Eurasia | Germany | *F. vesca* ssp. *vesca* | 47.599464; 10.056675 | | Plant | | Timo Hytonen | |
| 70 | Eurasia | Germany | *F. vesca* ssp. *vesca* | 47.635747; 9.605978 | | Plant | | Timo Hytonen | |
| 71 | Eurasia | Germany | *F. vesca* ssp. *vesca* | 50.5725; 9.15 | | Plant | | Timo Hytonen | |
| 72 | Eurasia | Germany | *F. vesca* ssp. *vesca* | 50.52868; 8.68424 | | Plant | | Timo Hytonen | |
| 73 | Eurasia | Germany | *F. vesca* ssp. *vesca* | 51.167015; 13.670816 | | Plant | | Timo Hytonen | |
| 74 | Eurasia | Germany | *F. vesca* ssp. *vesca* | 51.416239; 11.122212 | | Plant | | Timo Hytonen | |
| 75 | Eurasia | Germany | *F. vesca* ssp. *vesca* | 50.977175; 11.334845 | | Plant | | Timo Hytonen | |
| 76 | Eurasia | Germany | *F. vesca* ssp. *vesca* | 49.783954; 11.361754 | | Plant | | Timo Hytonen | |
| 77 | Eurasia | Germany | *F. vesca* ssp. *vesca* | 47.611794; 10.493202 | | Plant | | Timo Hytonen | |
| 78 | Eurasia | Germany | *F. vesca* ssp. *vesca* | 50.705148; 13.109319 | | Plant | | Timo Hytonen | |
| 79 | Eurasia | Germany | *F. vesca* ssp. *vesca* | 51.662847; 11.343787 | | Plant | | Timo Hytonen | |
| 80 | Eurasia | Germany | *F. vesca* ssp. *vesca* | 47.80331; 8.0369 | | Plant | | Timo Hytonen | |
| 81 | Eurasia | Italy | *F. vesca* ssp. *vesca* | 45.93633; 10.814 | | Plant | | Timo Hytonen | |
| 82 | Eurasia | Italy | *F. vesca* ssp. *vesca* | 46.01373; 11.050714 | | Plant | | Timo Hytonen | |
| 83 | Eurasia | Italy | *F. vesca* ssp. *vesca* | 46.034588; 11.01796 | | Plant | | Timo Hytonen | |
| 84 | Eurasia | Italy | *F. vesca* ssp. *vesca* | 46.027049; 10.998823 | | Plant | | Timo Hytonen | |
| 85 | Eurasia | Italy | *F. vesca* ssp. *vesca* | 45.87086; 10.7848 | | Plant | | Timo Hytonen | |
| 86 | Eurasia | Italy | *F. vesca* ssp. *vesca* | 45.88425; 10.80646 | | Plant | | Timo Hytonen | |
| 87 | Eurasia | Italy | *F. vesca* ssp. *vesca* | 45.75256; 10.86439 | | Plant | | Timo Hytonen | |
| 88 | Eurasia | Italy | *F. vesca* ssp. *vesca* | 45.82662; 10.96489 | | Plant | | Timo Hytonen | |
| 89 | Eurasia | Italy | *F. vesca* ssp. *vesca* | 45.9414; 10.814 | | Plant | | Timo Hytonen | |
| 90 | Eurasia | Italy | *F. vesca* ssp. *vesca* | 45.9415; 10.8141 | | Plant | | Timo Hytonen | |
| 91 | Eurasia | Italy | *F. vesca* ssp. *vesca* | 46.239797; 11.236043 | | Plant | | Timo Hytonen | |
| 92 | Eurasia | Italy | *F. vesca* ssp. *vesca* | 46.253001; 11.25391 | | Plant | | Timo Hytonen | |
| 93 | Eurasia | Italy | *F. vesca* ssp. *vesca* | 46.262205; 11.270901 | | Plant | | Timo Hytonen | |
| 94 | Eurasia | Italy | *F. vesca* ssp. *vesca* | 46.27501; 11.281761 | | Plant | | Timo Hytonen | |
| 95 | Eurasia | Italy | *F. vesca* ssp. *vesca* | 46.00564; 11.084477 | | Plant | | Timo Hytonen | |
| 96 | Eurasia | Lithuania | *F. vesca* ssp. *vesca* | 54.572901; 24.672245 | | Plant | | Timo Hytonen | |
| 97 | Eurasia | Lithuania | *F. vesca* ssp. *vesca* | 55.152649; 25.811486 | | Plant | | Timo Hytonen | |
| 98 | Eurasia | Lithuania | *F. vesca* ssp. *vesca* | 55.093794; 26.072359 | | Plant | | Timo Hytonen | |
| 99 | Eurasia | Lithuania | *F. vesca* ssp. *vesca* | 54.986036; 25.806088 | | Plant | | Timo Hytonen | |
| 100 | Eurasia | Lithuania | *F. vesca* ssp. *vesca* | 54.640067; 23.962387 | | Plant | | Timo Hytonen | |
| 101 | Eurasia | Sweden | *F. vesca* ssp. *vesca* | 58.13178; 11.60361 | | Plant | | Timo Hytonen | |
| 102 | Eurasia | Netherlands | *F. vesca* ssp. *vesca* | 50.865486; 5.690428 | | Plant | | Timo Hytonen | |
| 103 | Eurasia | Portugal | *F. vesca* ssp. *vesca* | 38.7923; -9.436918 | | Plant | | Timo Hytonen | |
| 104 | Eurasia | Portugal | *F. vesca* ssp. *vesca* | 38.792301; -9.436919 | | Plant | | Timo Hytonen | |
| 105 | Eurasia | Romania | *F. vesca* ssp. *vesca* | 46.777227; 23.551822 | | Plant | | Timo Hytonen | |
| 106 | Eurasia | Russia | *F. vesca* ssp. *vesca* | 51.94989; 85.944919 | | Plant | | Timo Hytonen | |
| 107 | Eurasia | Russia | *F. vesca* ssp. *vesca* | 55.631312; 85.959658 | | Plant | | Timo Hytonen | |
| 108 | Eurasia | Russia | *F. vesca* ssp. *vesca* | 52.179577; 104.342974 | | Plant | | Timo Hytonen | |
| 109 | Eurasia | Sweden | *F. vesca* ssp. *vesca* | 56.631802; 16.455866 | | Plant | | Timo Hytonen | |
| 110 | Eurasia | Sweden | *F. vesca* ssp. *vesca* | 59.8261; 16.886967 | | Plant | | Timo Hytonen | |
| 111 | Eurasia | Sweden | *F. vesca* ssp. *vesca* | 60.436867; 18.42595 | | Plant | | Timo Hytonen | |
| 112 | Eurasia | Sweden | *F. vesca* ssp. *vesca* | 60.365467; 17.28565 | | Plant | | Timo Hytonen | |
| 113 | Eurasia | Sweden | *F. vesca* ssp. *vesca* | 60.235767; 17.683667 | | Plant | | Timo Hytonen | |
| 114 | Eurasia | Sweden | *F. vesca* ssp. *vesca* | 60.1018; 18.343333 | | Plant | | Timo Hytonen | |
| 115 | Eurasia | Sweden | *F. vesca* ssp. *vesca* | 59.897633; 17.491667 | | Plant | | Timo Hytonen | |
| 116 | Eurasia | Sweden | *F. vesca* ssp. *vesca* | 59.758583; 18.0445 | | Plant | | Timo Hytonen | |
| 120 | Eurasia | Switzerland | *F. vesca* ssp. *vesca* | 46.411589; 8.133229 | | Plant | | Timo Hytonen | |
| 121 | Eurasia | UK | *F. vesca* ssp. *vesca* | 57.904881; -5.156961 | | Plant | | Timo Hytonen | |
| 122 | Eurasia | UK | *F. vesca* ssp. *vesca* | 51.484547; -1.026138 | | Plant | | Timo Hytonen | |
| 123 | Eurasia | UK | *F. vesca* ssp. *vesca* | 54.601389; -3.319662 | | Plant | | Timo Hytonen | |
| 124 | Eurasia | UK | *F. vesca* ssp. *vesca* | 54.726054; -3.217639 | | Plant | | Timo Hytonen | |
| 125 | Eurasia | UK | *F. vesca* ssp. *vesca* | 51.97842; -4.907069 | | Plant | | Timo Hytonen | |
| 126 | Eurasia | UK | *F. vesca* ssp. *vesca* | 57.918766; -4.423488 | | Plant | | Timo Hytonen | |
| 127 | Eurasia | UK | *F. vesca* ssp. *vesca* | 57.656325; -4.197719 | | Plant | | Timo Hytonen | |
| 128 | Eurasia | UK | *F. vesca* ssp. *vesca* | 54.565379; -3.136706 | | Plant | | Timo Hytonen | |
| 129 | Eurasia | UK | *F. vesca* ssp. *vesca* | 55.881703; -3.227013 | | Plant | | Timo Hytonen | |
| 130 | Eurasia | UK | *F. vesca* ssp. *vesca* | 51.484547; -1.026138 | | Plant | | Timo Hytonen | |
| 132 | Eurasia | Sweden | *F. vesca* ssp. *vesca* | 56.06324; 14.526432 | | Plant | | USDA GRIN (PI551841) | |
| 133 | Eurasia | Germany | *F. vesca* ssp. *vesca* | 47.96666667; 7.83333333 | | Plant | | USDA GRIN (PI616612) | |
| 134 | Eurasia | Russia | *F. vesca* ssp. *vesca* | 172km of Highway 34 | | Seed | | USDA GRIN (PI551891) | |
| 135 | Eurasia | Russia | *F. vesca* ssp. *vesca* | 54846054; 83.146982 | | Seed | | USDA GRIN (PI551892) | |
| 136 | Eurasia | Russia | *F. vesca* ssp. *vesca* | 54846055; 83.146983 | | Seed | | USDA GRIN (PI551890) | |
| 137 | Eurasia | Poland | *F. vesca* ssp. *vesca* | 53.43333; 16.16667 | | Seed | | USDA GRIN (PI551649) | |
| 138 | Eurasia | Italy | *F. vesca* ssp. *vesca* | 44.8; 7.08333 | | Seed | | USDA GRIN (PI602931) | |
| 139 | Eurasia | Bulgaria | *F. vesca* ssp. *vesca* | 41.95667; 24.12583 | | Seed | | USDA GRIN (PI616862) | |
| 140 | Eurasia | Bulgaria | *F. vesca* ssp. *vesca* | 41.99528; 24.79056 | | Seed | | USDA GRIN (PI616863) | |
| 141 | Eurasia | Bulgaria | *F. vesca* ssp. *vesca* | 41.66639; 24.41028 | | Seed | | USDA GRIN (PI616865) | |
| 142 | Eurasia | Bulgaria | *F. vesca* ssp. *vesca* | 42.80889; 24.63333 | Seed | | USDA GRIN (PI616866) | |  |
| 143 | Eurasia | Ukraine | *F. vesca* ssp. *vesca* | 49.30917; 33.22028 | | Seed | | USDA GRIN (PI635079) | |
| 144 | Eurasia | Albania | *F. vesca* ssp. *vesca* | 42.38723; 19.75715 | | Seed | | USDA GRIN (PI664466) | |
| 145 | Eurasia | Georgia | *F. vesca* ssp. *vesca* | 41.91851; 45.584026 | | Seed | | USDA GRIN (PI670253) | |
| 146 | Eurasia | Georgia | *F. vesca* ssp. *vesca* | 41.466589; 46.116873 | | Seed | | USDA GRIN (PI670254) | |
| 147 | Eurasia | Russia | *F. vesca* ssp. *vesca* | 50; 105 | | Plant | | USDA GRIN (PI616610) | |
| 148 | Eurasia | Bulgaria | *F. vesca* ssp. *vesca* | 42.86167; 24.52028 | | Seed | | USDA GRIN (PI616867) | |
| 149 | Eurasia | Germany | *F. vesca* ssp. *vesca* | 51; 11 | | Seed | | USDA GRIN (PI651546) | |
| 152 | Eurasia | Russia | *F. vesca* ssp. *vesca* | 50; 105 | | Plant | | USDA GRIN (PI616610) | |
| 153 | Eurasia | Finland | *F. vesca* ssp. *vesca* | 60.48673; 22.1534 | | Plant | | Timo Hytonen | |
| 154 | Eurasia | Finland | *F. vesca* ssp. *vesca* | 60.19488333; 21.33488333 | | Plant | | Timo Hytonen | |
| 155 | Eurasia | Finland | *F. vesca* ssp. *vesca* | 62.14489; 26.12041 | | Plant | | Timo Hytonen | |
| 156 | Eurasia | Finland | *F. vesca* ssp. *vesca* | 62.312; 27.534 | | Plant | | Timo Hytonen | |
| 157 | Eurasia | Finland | *F. vesca* ssp. *vesca* | 62.30807; 26.43993 | | Plant | | Timo Hytonen | |
| 158 | Eurasia | Finland | *F. vesca* ssp. *vesca* | 62.33517; 26.00523 | | Plant | | Timo Hytonen | |
| 159 | Eurasia | Finland | *F. vesca* ssp. *vesca* | 62.98699; 26.14083 | | Plant | | Timo Hytonen | |
| 160 | Eurasia | Finland | *F. vesca* ssp. *vesca* | 63.05; 25.82 | | Plant | | Timo Hytonen | |
| 161 | Eurasia | Finland | *F. vesca* ssp. *vesca* | 63.21271667; 28.20098333 | | Plant | | Timo Hytonen | |
| 162 | Eurasia | Finland | *F. vesca* ssp. *vesca* | 60.2; 21.4 | | Plant | | Timo Hytonen | |
| 163 | Eurasia | Finland | *F. vesca* ssp. *vesca* | 63.31833333; 25.55075 | | Plant | | Timo Hytonen | |
| 164 | Eurasia | Finland | *F. vesca* ssp. *vesca* | 62.6034; 23.86053333 | | Plant | | Timo Hytonen | |
| 165 | Eurasia | Finland | *F. vesca* ssp. *vesca* | 62.86643333; 27.6723 | | Plant | | Timo Hytonen | |
| 166 | Eurasia | Finland | *F. vesca* ssp. *vesca* | 63.31341667; 29.89443333 | | Plant | | Timo Hytonen | |
| 167 | Eurasia | Finland | *F. vesca* ssp. *vesca* | 62.48023333; 24.3899 | | Plant | | Timo Hytonen | |
| 168 | Eurasia | Finland | *F. vesca* ssp. *vesca* | 62.9; 28.2 | | Plant | | Timo Hytonen | |
| 169 | Eurasia | Finland | *F. vesca* ssp. *vesca* | 60.15; 21.59 | | Plant | | Timo Hytonen | |
| 170 | Eurasia | Finland | *F. vesca* ssp. *vesca* | 63.70208333; 28.86095 | | Plant | | Timo Hytonen | |
| 171 | Eurasia | Finland | *F. vesca* ssp. *vesca* | 62.33403333; 21.19113333 | | Plant | | Timo Hytonen | |
| 172 | Eurasia | Finland | *F. vesca* ssp. *vesca* | 61.19863; 26.85067 | | Plant | | Timo Hytonen | |
| 173 | Eurasia | Finland | *F. vesca* ssp. *vesca* | 60.9789; 25.9305 | | Plant | | Timo Hytonen | |
| 174 | Eurasia | Finland | *F. vesca* ssp. *vesca* | 60.35; 25.24 | | Plant | | Timo Hytonen | |
| 175 | Eurasia | Finland | *F. vesca* ssp. *vesca* | 60.53665; 21.87658 | | Plant | | Timo Hytonen | |
| 176 | Eurasia | Finland | *F. vesca* ssp. *vesca* | 61.58244; 26.01867 | | Plant | | Timo Hytonen | |
| 177 | Eurasia | Finland | *F. vesca* ssp. *vesca* | 67.77957; 29.6048 | | Plant | | Timo Hytonen | |
| 178 | Eurasia | Finland | *F. vesca* ssp. *vesca* | 67.792; 29.4471 | | Plant | | Timo Hytonen | |
| 179 | Eurasia | Finland | *F. vesca* ssp. *vesca* | 65.2101; 25.2777 | | Plant | | Timo Hytonen | |
| 180 | Eurasia | Finland | *F. vesca* ssp. *vesca* | 65.21; 21.49 | | Plant | | Timo Hytonen | |
| 181 | Eurasia | Finland | *F. vesca* ssp. *vesca* | 65.29; 25.38 | | Plant | | Timo Hytonen | |
| 182 | Eurasia | Finland | *F. vesca* ssp. *vesca* | 65.6751; 24.5182 | | Plant | | Timo Hytonen | |
| 183 | Eurasia | Finland | *F. vesca* ssp. *vesca* | 66.51667; 25.7875 | | Plant | | Timo Hytonen | |
| 184 | Eurasia | Finland | *F. vesca* ssp. *vesca* | 65.86067; 24.95476 | | Plant | | Timo Hytonen | |
| 185 | Eurasia | Finland | *F. vesca* ssp. *vesca* | 65.77; 24.16 | | Plant | | Timo Hytonen | |
| 186 | Eurasia | Finland | *F. vesca* ssp. *vesca* | 65.81893; 24.5171 | | Plant | | Timo Hytonen | |
| 187 | Eurasia | Finland | *F. vesca* ssp. *vesca* | 61.28081667; 26.8045 | | Plant | | Timo Hytonen | |
| 188 | Eurasia | Finland | *F. vesca* ssp. *vesca* | 66.46074; 24.76848 | | Plant | | Timo Hytonen | |
| 189 | Eurasia | Finland | *F. vesca* ssp. *vesca* | 61.6938; 27.70831667 | | Plant | | Timo Hytonen | |
| 190 | Eurasia | Finland | *F. vesca* ssp. *vesca* | 61.53375; 23.39193333 | | Plant | | Timo Hytonen | |
| 191 | Eurasia | Finland | *F. vesca* ssp. *vesca* | 61.35583333; 26.59985 | | Plant | | Timo Hytonen | |
| 192 | Eurasia | Finland | *F. vesca* ssp. *vesca* | 60.71958333; 24.68238333 | | Plant | | Timo Hytonen | |
| 193 | Eurasia | Finland | *F. vesca* ssp. *vesca* | 60.34306667; 25.67945 | | Plant | | Timo Hytonen | |
| 194 | Eurasia | Finland | *F. vesca* ssp. *vesca* | 60.47753333; 24.84441667 | | Plant | | Timo Hytonen | |
| 195 | Eurasia | Finland | *F. vesca* ssp. *vesca* | 60.72617; 21.90933 | | Plant | | Timo Hytonen | |
| 196 | Eurasia | Finland | *F. vesca* ssp. *vesca* | 61.16405; 23.78073333 | | Plant | | Timo Hytonen | |
| 197 | Eurasia | Finland | *F. vesca* ssp. *vesca* | 60.40511667; 25.15616667 | | Plant | | Timo Hytonen | |
| 198 | Eurasia | Finland | *F. vesca* ssp. *vesca* | 60.80517; 23.757 | | Plant | | Timo Hytonen | |
| 199 | Eurasia | Finland | *F. vesca* ssp. *vesca* | 60.40386667; 23.12518333 | | Plant | | Timo Hytonen | |
| 200 | Eurasia | Finland | *F. vesca* ssp. *vesca* | 61.8788; 21.46825 | | Plant | | Timo Hytonen | |
| 201 | Eurasia | Finland | *F. vesca* ssp. *vesca* | 61.79486667; 24.35845 | | Plant | | Timo Hytonen | |
| 202 | Eurasia | Finland | *F. vesca* ssp. *vesca* | 60.10611667; 23.67818333 | | Plant | | Timo Hytonen | |
| 203 | Eurasia | Finland | *F. vesca* ssp. *vesca* | 60.06728333; 23.28788333 | | Plant | | Timo Hytonen | |
| 204 | Eurasia | Finland | *F. vesca* ssp. *vesca* | 60.2076; 23.80655 | | Plant | | Timo Hytonen | |
| 205 | Eurasia | Finland | *F. vesca* ssp. *vesca* | 60.46513333; 25.52535 | | Plant | | Timo Hytonen | |
| 206 | Eurasia | Finland | *F. vesca* ssp. *vesca* | 61.8; 29.3 | | Plant | | Timo Hytonen | |
| 207 | Eurasia | Finland | *F. vesca* ssp. *vesca* | 60.3706; 22.97958333 | | Plant | | Timo Hytonen | |
| 208 | Eurasia | Iceland | *F. vesca* ssp. *vesca* | 60.08241667; 24.15918333 | | Plant | | J. H. Hallsson & H. S. Hilmarsson | |
| 209 | Eurasia | Iceland | *F. vesca* ssp. *vesca* | 64.799766; 21.254027 | | Plant | | J. H. Hallsson & H. S. Hilmarsson | |
| 210 | Eurasia | Iceland | *F. vesca* ssp. *vesca* | 64.57149; 21.59908 | | Plant | | J. H. Hallsson & H. S. Hilmarsson | |
| 211 | Eurasia | Iceland | *F. vesca* ssp. *vesca* | 64.75928; 21.59377 | | Plant | | J. H. Hallsson & H. S. Hilmarsson | |
| 212 | Eurasia | Iceland | *F. vesca* ssp. *vesca* | 64.83845; 21.35098 | | Plant | | J. H. Hallsson & H. S. Hilmarsson | |
| 213 | Eurasia | Iceland | *F. vesca* ssp. *vesca* | 65.95007; 19.48785 | | Plant | | J. H. Hallsson & H. S. Hilmarsson | |
| 214 | Eurasia | Iceland | *F. vesca* ssp. *vesca* | 65.950069; -19.487877 | | Plant | | J. H. Hallsson & H. S. Hilmarsson | |
| 215 | Eurasia | Iceland | *F. vesca* ssp. *vesca* | 63.992617; -19.956233 | | Plant | | J. H. Hallsson & H. S. Hilmarsson | |
| 216 | Eurasia | Iceland | *F. vesca* ssp. *vesca* | 63.96063; -22.398191 | | Plant | | J. H. Hallsson & H. S. Hilmarsson | |
| 217 | Eurasia | Iceland | *F. vesca* ssp. *vesca* | 63.960631; -22.398192 | | Plant | | J. H. Hallsson & H. S. Hilmarsson | |
| 218 | Eurasia | Iceland | *F. vesca* ssp. *vesca* | 63.960632; -22.398193 | | Plant | | J. H. Hallsson & H. S. Hilmarsson | |
| 219 | Eurasia | Iceland | *F. vesca* ssp. *vesca* | 65.671034; -18.043221 | | Plant | | J. H. Hallsson & H. S. Hilmarsson | |
| 220 | Eurasia | Iceland | *F. vesca* ssp. *vesca* | 65.671035; -18.043222 | | Plant | | J. H. Hallsson & H. S. Hilmarsson | |
| 221 | Eurasia | Iceland | *F. vesca* ssp. *vesca* | 63645833; -18.505583 | | Plant | | J. H. Hallsson & H. S. Hilmarsson | |
| 222 | Eurasia | Iceland | *F. vesca* ssp. *vesca* | 63.8372; -18.050467 | | Plant | | J. H. Hallsson & H. S. Hilmarsson | |
| 223 | Eurasia | Iceland | *F. vesca* ssp. *vesca* | 63.8372; -18.050467 | | Plant | | J. H. Hallsson & H. S. Hilmarsson | |
| 224 | Eurasia | Iceland | *F. vesca* ssp. *vesca* | 63.947267; -17.64025 | | Plant | | J. H. Hallsson & H. S. Hilmarsson | |
| 225 | Eurasia | Iceland | *F. vesca* ssp. *vesca* | 63.947267; -17.64025 | | Plant | | J. H. Hallsson & H. S. Hilmarsson | |
| 226 | Eurasia | Iceland | *F. vesca* ssp. *vesca* | 63.972315; -16.839655 | | Plant | | J. H. Hallsson & H. S. Hilmarsson | |
| 227 | Eurasia | Iceland | *F. vesca* ssp. *vesca* | 63.972316; -16.839656 | | Plant | | J. H. Hallsson & H. S. Hilmarsson | |
| 228 | Eurasia | Iceland | *F. vesca* ssp. *vesca* | 65.5616; -18.1062 | | Plant | | J. H. Hallsson & H. S. Hilmarsson | |
| 229 | Eurasia | Iceland | *F. vesca* ssp. *vesca* | 65.5616; -18.1062 | | Plant | | J. H. Hallsson & H. S. Hilmarsson | |
| 230 | Eurasia | Iceland | *F. vesca* ssp. *vesca* | 65.5616; -18.1062 | | Plant | | J. H. Hallsson & H. S. Hilmarsson | |
| 231 | Eurasia | Iceland | *F. vesca* ssp. *vesca* | 65.5616; -18.1062 | | Plant | | J. H. Hallsson & H. S. Hilmarsson | |
| 232 | Eurasia | Iceland | *F. vesca* ssp. *vesca* | 65.999876; -16.510578 | | Plant | | J. H. Hallsson & H. S. Hilmarsson | |
| 233 | Eurasia | Iceland | *F. vesca* ssp. *vesca* | 65.998511; -16.512318 | | Plant | | J. H. Hallsson & H. S. Hilmarsson | |
| 234 | Eurasia | Iceland | *F. vesca* ssp. *vesca* | 65.998511; -16.512318 | | Plant | | J. H. Hallsson & H. S. Hilmarsson | |
| 235 | Eurasia | Iceland | *F. vesca* ssp. *vesca* | 66.002366; -16.517227 | | Plant | | J. H. Hallsson & H. S. Hilmarsson | |
| 236 | Eurasia | Iceland | *F. vesca* ssp. *vesca* | 63.990933; -19.963683 | | Plant | | J. H. Hallsson & H. S. Hilmarsson | |
| 237 | Eurasia | Iceland | *F. vesca* ssp. *vesca* | 64.00795; -19.881 | | Plant | | J. H. Hallsson & H. S. Hilmarsson | |
| 238 | Eurasia | Iceland | *F. vesca* ssp. *vesca* | 64.069533; -19.851433 | | Plant | | J. H. Hallsson & H. S. Hilmarsson | |
| 239 | Eurasia | Iceland | *F. vesca* ssp. *vesca* | 64.068367; -19.846767 | | Plant | | J. H. Hallsson & H. S. Hilmarsson | |
| 240 | Eurasia | Iceland | *F. vesca* ssp. *vesca* | 64.06785; -19.847283 | | Plant | | J. H. Hallsson & H. S. Hilmarsson | |
| 241 | Eurasia | Iceland | *F. vesca* ssp. *vesca* | 64.058783; -19.860517 | | Plant | | J. H. Hallsson & H. S. Hilmarsson | |
| 242 | Eurasia | Iceland | *F. vesca* ssp. *vesca* | 64.059317; -19.858367 | | Plant | | J. H. Hallsson & H. S. Hilmarsson | |
| 243 | Eurasia | Iceland | *F. vesca* ssp. *vesca* | 64.059567; -19.8573 | | Plant | | J. H. Hallsson & H. S. Hilmarsson | |
| 244 | Eurasia | Iceland | *F. vesca* ssp. *vesca* | 64.060117; -19.85225 | | Plant | | J. H. Hallsson & H. S. Hilmarsson | |
| 245 | Eurasia | Iceland | *F. vesca* ssp. *vesca* | 64.060118; -19.85225 | | Plant | | J. H. Hallsson & H. S. Hilmarsson | |
| 246 | Eurasia | Iceland | *F. vesca* ssp. *vesca* | 64.062217; -19.847217 | | Plant | | J. H. Hallsson & H. S. Hilmarsson | |
| 247 | Eurasia | Iceland | *F. vesca* ssp. *vesca* | 64.064083; -19.8394 | | Plant | | J. H. Hallsson & H. S. Hilmarsson | |
| 248 | Eurasia | Iceland | *F. vesca* ssp. *vesca* | 64.064367; -19.83455 | | Plant | | J. H. Hallsson & H. S. Hilmarsson | |
| 249 | Eurasia | Iceland | *F. vesca* ssp. *vesca* | 64.064467; -19.825283 | | Plant | | J. H. Hallsson & H. S. Hilmarsson | |
| 250 | Eurasia | Iceland | *F. vesca* ssp. *vesca* | 64.521583; -21.436167 | | Plant | | J. H. Hallsson & H. S. Hilmarsson | |
| 251 | Eurasia | Iceland | *F. vesca* ssp. *vesca* | 64.521567; -21.431283 | | Plant | | J. H. Hallsson & H. S. Hilmarsson | |
| 252 | Eurasia | Iceland | *F. vesca* ssp. *vesca* | 65.34375; -20.214383 | | Plant | | J. H. Hallsson & H. S. Hilmarsson | |
| 253 | Eurasia | Iceland | *F. vesca* ssp. *vesca* | 64.735431; -22.041482 | | Plant | | J. H. Hallsson & H. S. Hilmarsson | |
| 254 | Eurasia | Iceland | *F. vesca* ssp. *vesca* | 64.735431; -22041482 | | Plant | | J. H. Hallsson & H. S. Hilmarsson | |
| 255 | Eurasia | Iceland | *F. vesca* ssp. *vesca* | 64.760397; -22.133825 | | Plant | | J. H. Hallsson & H. S. Hilmarsson | |
| 256 | Eurasia | Iceland | *F. vesca* ssp. *vesca* | 65.756178; -19.538124 | | Plant | | J. H. Hallsson & H. S. Hilmarsson | |
| 257 | Eurasia | Iceland | *F. vesca* ssp. *vesca* | 64.006483; -20.975117 | | Plant | | J. H. Hallsson & H. S. Hilmarsson | |
| 258 | Eurasia | Iceland | *F. vesca* ssp. *vesca* | 65.756178; -19.538124 | | Plant | | J. H. Hallsson & H. S. Hilmarsson | |
| 259 | Eurasia | Iceland | *F. vesca* ssp. *vesca* | 64.754888; -21.599182 | | Plant | | J. H. Hallsson & H. S. Hilmarsson | |
| 260 | Eurasia | Iceland | *F. vesca* ssp. *vesca* | 64.754824; -21.599076 | | Plant | | J. H. Hallsson & H. S. Hilmarsson | |
| 261 | Eurasia | Iceland | *F. vesca* ssp. *vesca* | 64.972212; -15.134431 | | Plant | | J. H. Hallsson & H. S. Hilmarsson | |
| 262 | Eurasia | Norway | *F. vesca* ssp. *vesca* | 69.93955; 23.09714 | | Plant | | Timo Hytonen | |
| 263 | Eurasia | Norway | *F. vesca* ssp. *vesca* | 69.9166; 23.0001 | | Plant | | Timo Hytonen | |
| 264 | Eurasia | Norway | *F. vesca* ssp. *vesca* | 70.18481; 23.37255 | | Plant | | Timo Hytonen | |
| 265 | Eurasia | Norway | *F. vesca* ssp. *vesca* | 69.52483; 18.37517 | | Plant | | Timo Hytonen | |
| 266 | Eurasia | Norway | *F. vesca* ssp. *vesca* | 69.52483; 18.37517 | | Plant | | Timo Hytonen | |
| 267 | Eurasia | Norway | *F. vesca* ssp. *vesca* | 69.52483; 18.37517 | | Plant | | Timo Hytonen | |
| 268 | Eurasia | Norway | *F. vesca* ssp. *vesca* | 69.522; 18.15933 | | Plant | | Timo Hytonen | |
| 269 | Eurasia | Norway | *F. vesca* ssp. *vesca* | 69.53586; 20.38052 | | Plant | | Timo Hytonen | |
| 270 | Eurasia | Norway | *F. vesca* ssp. *vesca* | 69.53586; 20.38052 | | Plant | | Timo Hytonen | |
| 271 | Eurasia | Norway | *F. vesca* ssp. *vesca* | 69.52739; 20.37237 | | Plant | | Timo Hytonen | |
| 272 | Eurasia | Norway | *F. vesca* ssp. *vesca* | 69.4451; 20.949 | | Plant | | Timo Hytonen | |
| 273 | Eurasia | Norway | *F. vesca* ssp. *vesca* | 69.42555; 20.97149 | | Plant | | Timo Hytonen | |
| 274 | Eurasia | Finland | *F. vesca* ssp. *vesca* | 60.35601667; 22.96513333 | | Plant | | Timo Hytonen | |
| 275 | Eurasia | Norway | *F. vesca* ssp. *vesca* | 69.90233; 21.89166 | | Plant | | Timo Hytonen | |
| 276 | Eurasia | Norway | *F. vesca* ssp. *vesca* | 70.0301; 22.0653 | | Plant | | Timo Hytonen | |
| 277 | Eurasia | Norway | *F. vesca* ssp. *vesca* | 69.9395; 23.09643 | | Plant | | Timo Hytonen | |
| 278 | Eurasia | Norway | *F. vesca* ssp. *vesca* | 70.02267; 22.01734 | | Plant | | Timo Hytonen | |
| 279 | Eurasia | Norway | *F. vesca* ssp. *vesca* | 70.16708; 24.7561 | | Plant | | Timo Hytonen | |
| 280 | Eurasia | Norway | *F. vesca* ssp. *vesca* | 69.85366; 25.051 | | Plant | | Timo Hytonen | |
| 281 | Eurasia | Norway | *F. vesca* ssp. *vesca* | 70.3216; 28.16984 | | Plant | | Timo Hytonen | |
| 282 | Eurasia | Norway | *F. vesca* ssp. *vesca* | 64.5; 21.49 | | Plant | | Timo Hytonen | |
| 283 | Eurasia | Norway | *F. vesca* ssp. *vesca* | -; - | | Plant | | Timo Hytonen | |
| 284 | Eurasia | Norway | *F. vesca* ssp. *vesca* | 59.67; 27.668 | | Plant | | Timo Hytonen | |
| 285 | Eurasia | Norway | *F. vesca* ssp. *vesca* | 69.93955; 23.09714 | | Plant | | Timo Hytonen | |
| 286 | Eurasia | Norway | *F. vesca* ssp. *vesca* | 69.95136; 23.05133 | | Plant | | Timo Hytonen | |
| 287 | Eurasia | Norway | *F. vesca* ssp. *vesca* | 70.0226; 23.55951 | | Plant | | Timo Hytonen | |
| 288 | Eurasia | Norway | *F. vesca* ssp. *vesca* | 70.02645; 23.49423 | | Plant | | Timo Hytonen | |
| 289 | Eurasia | Norway | *F. vesca* ssp. *vesca* | 70.02727; 23.38741 | | Plant | | Timo Hytonen | |
| 290 | Eurasia | Norway | *F. vesca* ssp. *vesca* | 70.03244; 23.40124 | | Plant | | Timo Hytonen | |
| 291 | Eurasia | Norway | *F. vesca* ssp. *vesca* | 70.15852; 23.27812 | | Plant | | Timo Hytonen | |
| 292 | Eurasia | Finland | *F. vesca* ssp. *vesca* | 59.8428; 23.24463333 | | Plant | | Timo Hytonen | |
| 295 | Eurasia | Kazakhstan | *F. vesca* ssp. *vesca* | 43.09319; 77.045188 | | Seed | | USDA GRIN (PI552274) | |
| 296 | Eurasia | Kazakhstan | *F. vesca* ssp. *vesca* | 43.19014; 77.16448 | | Seed | | USDA GRIN (PI552273) | |
| 297 | Eurasia | Kyrgyzstan | *F. vesca* ssp. *vesca* | 42.56359; 74.66125 | | Seed | | USDA GRIN (PI660760) | |
| 298 | Japan | Japan | *F. vesca* ssp. *vesca* | 43.0653; 141.34409 | | Seed | | USDA GRIN (PI660771) | |
| 299 | Japan | Japan | *F. vesca* ssp. *vesca* | 42.73721; 140.65747 | | Seed | | USDA GRIN (PI664377) | |
| 150 | Eurasia* | Sweden | *F. vesca* ssp. *vesca* f. Alba | 56063240; 14.526432 | | Plant | | USDA GRIN (PI551841) | |
| 151 | Eurasia* | Sweden | *F. vesca* ssp. *vesca* f. Alba | 59.809635; 17.729858 | | Plant | | USDA GRIN (PI551909) | |
| 293 | Eurasia/outgroup | Sweden | *F*. *viridis* | 57.33333; 18.66667 | | Seed | | USDA GRIN (PI 616857) | |
| 117 | Eurasia* | Russia | *F. vesca* ssp. *vesca* | 56.498062; 84.896021 | | Seed | | Botanical garden, Novosibrisk | |
| 118 | Eurasia* | Russia | *F. vesca* ssp. *vesca* | 56.498062; 84.896022 | | Seed | | Botanical garden, Novosibrisk | |
| 119 | Eurasia* | Russia | *F. vesca* ssp. *vesca* | 56.498062; 84.896023 | | Seed | | Botanical garden, Novosibrisk | |
| 7 | America | USA-South Dakota | *F. vesca* ssp. *americana* | 43.83333; -103.5 | | Plant | | USDA GRIN (PI 551881) | |
| 8 | America | USA-New Hampshire | *F. vesca* ssp. *americana* | 43.10575; -71.18295 | | Plant | | USDA GRIN (PI 657856) | |
| 23 | America | USA-Nebraska | *F. vesca* ssp. *americana* | 42.61686; -103.19389 | | Plant | | USDA GRIN (PI 651579) | |
| 26 | America | Canada-Ontario | *F. vesca* ssp. *americana* | 44.9125; -76.48889 | | Plant | | USDA GRIN (PI 616728) | |
| 28 | America | USA-New York | *F. vesca* ssp. *americana* | 42.7602; -74.1069 | | Plant | | USDA GRIN (PI 552286) | |
| 39 | America | USA-Iowa | *F. vesca* ssp. *americana* | 42.33333; -94.16667 | | Seed | | USDA GRIN (PI 551519) | |
| 1 | America | Canada | *F. vesca* ssp. bracteata 'Pacific' | 50.7875; -126.502778 | | Plant | | Can. clonal genebank (CN99586) | |
| 2 | America | Canada | *F. vesca* ssp. bracteata 'Pacific' | 50.787501; -126.502779 | | Plant | | Can. clonal genebank (CN99582) | |
| 3 | America | Canada | *F. vesca* ssp. bracteata 'Pacific' | 50.119444; -125.075 | | Plant | | Can. clonal genebank (CN99574) | |
| 4 | America | Canada | *F. vesca* ssp. bracteata 'Pacific' | 50.119445; -125.075001 | | Plant | | Can. clonal genebank (CN99570) | |
| 13 | America | USA-Oregon | *F. vesca* ssp. bracteata 'Pacific' | 44.83333; -122.33333 | | Plant | | USDA GRIN (PI 552291) | |
| 17 | America | Canada-British Columbia | *F. vesca* ssp. bracteata 'Pacific' | 49.15; -124.5 | | Plant | | USDA GRIN (PI 616651) | |
| 20 | America | USA-Oregon | *F. vesca* ssp. bracteata 'Pacific' | 44.488444; -123.537417 | | Plant | | USDA GRIN (PI 664465) | |
| 25 | America | USA-Oregon | *F. vesca* ssp. bracteata 'Pacific' | 44.85358; -122.6641 | | Plant | | USDA GRIN (PI 664389) | |
| 27 | America | USA-Oregon | *F. vesca* ssp. bracteata 'Pacific' | 45.3725; -121.8226 | | Plant | | USDA GRIN (PI 664413) | |
| 29 | America | USA-Oregon | *F. vesca* ssp. bracteata 'Pacific' | 42.16667; -123.5 | | Seed | | USDA GRIN (PI 551783) | |
| 31 | America | USA-Oregon | *F. vesca* ssp. bracteata 'Pacific' | 43.5; -121.5 | | Plant | | USDA GRIN (PI 551791) | |
| 32 | America | USA-Oregon | *F. vesca* ssp. bracteata 'Pacific' | 42.5; -123.16667 | | Plant | | USDA GRIN (PI 551784) | |
| 34 | America | Canada-British Columbia | *F. vesca* ssp. bracteata 'Pacific' | 53.90578; -122.55703 | | Seed | | USDA GRIN (PI 660764) | |
| 10 | America | USA-New Mexico | *F. vesca* ssp. bracteata 'Rocky Mts' | 35.7025; -105.68639 | | Plant | | USDA GRIN (PI 637947) | |
| 11 | America | Mexico | *F. vesca* ssp. bracteata 'Rocky Mts' | 24.5; -105 | | Plant | | USDA GRIN (PI 442362) | |
| 14 | America | USA-Montana | *F. vesca* ssp. bracteata 'Rocky Mts' | 46.03138; -114.17138 | | Plant | | USDA GRIN (PI 551525) | |
| 19 | America | USA-Idaho | *F. vesca* ssp. bracteata 'Rocky Mts' | 48.53761; -116.35661 | | Plant | | USDA GRIN (PI 551646) | |
| 22 | America | USA-Colorado | *F. vesca* ssp. bracteata 'Rocky Mts' | 39.69315; -105.50125 | | Plant | | USDA GRIN (PI 657860) | |
| 30 | America | USA-Arizona | *F. vesca* ssp. bracteata 'Rocky Mts' | 31.882; -109.2841 | | Plant | | USDA GRIN (PI 637952) | |
| 40 | America | USA-Idaho | *F. vesca* ssp. bracteata 'Rocky Mts' | 42.16257; -111.64625 | | Seed | | USDA GRIN (PI 651550) | |
| 9 | America | USA-California | *F. vesca* ssp. c*alifornica* | 39.24202; -123.51987 | | Plant (A) | | USDA GRIN (PI 660765) | |
| 24 | America | USA-California | *F. vesca* ssp. c*alifornica* | 39.24202; -123.51987 | | Plant (B) | | USDA GRIN (PI 660765) | |
| 35 | America | USA-California | *F. vesca* ssp. c*alifornica* | 36.54275; -117.468658 | | Plant | | USDA GRIN (PI 27089) | |
| 131 | America | USA-California | *F. vesca* ssp. c*alifornica* | 38.44334; -123.12586 | | Plant | | USDA GRIN (PI 551723) | |
| 6 | America | USA-New Hampshire | *F. vesca* ssp. *vesca* | 43.1278; -70.9325 | | Plant | | USDA GRIN (PI 552287) | |
| 15 | America | USA-Massachusetts | *F. vesca* ssp. *vesca* | 42.6849; 71.4942 | | Plant | | USDA GRIN (PI 552243) | |
| 16 | America | USA-Kentucky | *F. vesca* ssp. *vesca* | 36.9894; -84.5987 | | Plant | | USDA GRIN (PI 616575) | |
| 18 | America | USA-New Hampshire | *F. vesca* ssp. *vesca* | 44.4515; -71.5784 | | Plant | | USDA GRIN (PI 552245) | |
| 21 | America | USA-Massachusetts | *F. vesca* ssp. *vesca* | 42.6849; 71.4942 | | Plant | | USDA GRIN (PI 552248) | |
| 33 | America | Bolivia | *F. vesca* ssp. *vesca* | -17.864108; -63.885144 | | Plant | | USDA GRIN (PI 616673) | |
| 36 | America | Canada-Nova Scotia | *F. vesca* ssp. *vesca* | 45.07217; -64.507303 | | Plant | | USDA GRIN (PI 666642) | |
| 38 | America | Canada-British Columbia | *F. vesca* ssp. *vesca* | 53.27223; -120.06457 | | Seed | | USDA GRIN (PI 660763) | |
| 37 | America* | USA-Maryland | *F.* *vesca* ssp. *vesca* H4 / alba / f7 | -; - | | Plant | | USDA GRIN (PI 664444) | |
| 300 | America* | USA-Hawaii | *F.* *vesca* ssp. *vesca* Hawaii | 19.4453; -155.2851 | | Seed | | USDA GRIN (PI 664385) | |

*Grouped as cultivars.
