# Supplementary material for: Population genetic analysis of a global collection of Fragaria vesca using microsatellite markers
Source: PLoS One. 2017 Aug 30;12(8):e0183384. doi: 10.1371/journal.pone.0183384 (PMC5576660; doi:10.1371/journal.pone.0183384)
Supplement: S2 Table — Including marker name, sequence of forward and reverse primers, GenBank accession number (Acc. no.), pattern of repeats (Repeat), chromosome location (Chr.), the linkage position (Linkage), and species name. (DOCX) [file pone.0183384.s004.docx]

**S2 Table. Information on microsatellite markers used.**

|  | **Marker name** | **Acc.nr.** | **Type** | **Repeat** | **Chr., position** | **Reference** |
| --- | --- | --- | --- | --- | --- | --- |
| 1 | FVES0109 | AM901603 | EST-SSR | AG | Fvb1, 13 109 991 | Rousseau-Gueutin et al., 2010 |
| 2 | FVES0989 | EX662684 | EST-SSR | GGC | Fvb1, 13 495 042 | Rivarola et al., 2011. |
| 3 | FVES2950 | EX662228 | EST-SSR | AAC | Fvb1, 17 037 047 | Rivarola et al., 2011. |
| 4 | FVES3100 | DY675601 | EST-SSR | GGA | Fvb1, 3 105 465 | Rivarola et al., 2011. |
| 5 | FVES1877 | DQ830740 | EST-SSR | AATG | Fvb1, 4 092 938 | Direct submission: Oosumi and Shulaev, 2006. |
| 6 | FVES3330 | DY671364 | EST-SSR | AAG | Fvb1, 406 630 | Rivarola et al., 2011. |
| 7 | FVES1621 | EX666393 | EST-SSR | GGC | Fvb1, 5 938 104 | Rivarola et al., 2011. |
| 8 | FVES0459 | EX677809 | EST-SSR | AAG | Fvb1, 6 184 610 | Rivarola et al., 2011. |
| 9 | FVES1201 | DY667184 | EST-SSR | GGT | Fvb1, 7 221 482 | Rivarola et al., 2011. |
| 10 | FAES0479 | CO817128 | EST-SSR | GGC | Fvb1, 7 519 621 | Direct submission: Folta et al. 2004. |
| 11 | FVES1907 | EX687547 | EST-SSR | AAAG | Fvb1, 7014881 | Rivarola et al., 2011. |
| 12 | FAES0376 | CO378698 | EST-SSR | AATC | Fvb2, 1 123 162 | Direct submission: Carbone et al., 2004. |
| 13 | FVES1362(G) | DY671439 | EST-SSR | AAG | Fvb2, 15 451 150 | Rivarola et al., 2011. |
| 14 | FVES1160 | DY668745 | EST-SSR | GGA | Fvb2, 188 425 | Rivarola et al., 2011. |
| 15 | FAES0293 | CO817011 | EST-SSR | AAG | Fvb2, 26 102 277 | Direct submission: Folta et al. 2004. |
| 16 | FVES1230 | CX309678 | EST-SSR | AAG | Fvb2, 26 430 877 | Direct submission: Shulaev and Slovin, 2004. |
| 17 | FVES2661 | EX675599 | EST-SSR | ATC | Fvb2, 28 017 151 | Rivarola et al., 2011. |
| 18 | FAES0465 | CO817625 | EST-SSR | AAG | Fvb2, 4 130 884 | Direct submission: Folta et al. 2004. |
| 19 | RosCOS1238 | NA | EST-SSR | NA | Fvb2, 6 110 542 | Cabrera et al., 2009. |
| 20 | FVES0480 | DY669565 | EST-SSR | AAAT | Fvb3, 10 021 328 | Rivarola et al., 2011. |
| 21 | FVES2999 | EX660314 | EST-SSR | GGA | Fvb3, 15 110 397 | Rivarola et al., 2011. |
| 22 | FVES2300(B) | EX687556 | EST-SSR | GGT | Fvb3, 18 183 304 | Rivarola et al., 2011. |
| 23 | FVES1711 | DY674809 | EST-SSR | GGA | Fvb3, 2 704 129 | Rivarola et al., 2011. |
| 24 | FVES1224 | CX661936 | EST-SSR | AAG | Fvb3, 2 814 719 | Direct submission: Shulaev and Slovin, 2004. |
| 25 | FVES1156 | DY668442 | EST-SSR | ATC | Fvb3, 3 358 493 | Rivarola et al., 2011. |
| 26 | FVES1213 | DV440121 | EST-SSR | AGC | Fvb3, 4 166 818 | Direct submission: Brese et al., 2005. |
| 27 | FVES0960 | EX667577 | EST-SSR | AGC | Fvb3, 6 739 743 | Rivarola et al., 2011. |
| 28 | FVES1313 | EX670488 | EST-SSR | AAG | Fvb3, 7 985 642 | Rivarola et al., 2011. |
| 29 | FVES1793 | DY670278 | EST-SSR | AAT | Fvb3, 764 610 | Rivarola et al., 2011. |
| 30 | FVES0577 | EX674904 | EST-SSR | ACG | Fvb4, 1 078 578 | Rivarola et al., 2011. |
| 31 | FVES2882 | EX665774 | EST-SSR | GGA | Fvb4, 14 411 361 | Rivarola et al., 2011. |
| 32 | FVES1356 | DY673855 | EST-SSR | AAG | Fvb4, 15 466 483 | Rivarola et al., 2011. |
| 33 | FVES0007 | DY672378 | EST-SSR | AAG | Fvb4, 18 341 005 | Rivarola et al., 2011. |
| 34 | FVES0567 | EX677405 | EST-SSR | GGC | Fvb4, 20 976 966 | Rivarola et al., 2011. |
| 35 | FVES2281 | EX687013 | EST-SSR | AAG | Fvb4, 22 392 247 | Rivarola et al., 2011. |
| 36 | FVES2235 | AJ001449 | EST-SSR | AAT | Fvb4, 29 894 990 | Direct submission: Nam et al., 1997 |
| 37 | FVES1031 | EX657459 | EST-SSR | AGC | Fvb4, 30 806 105 | Rivarola et al., 2011. |
| 38 | FAES0581 | CO380995 | EST-SSR | AGC | Fvb4, 31 330 746 | Direct submission: Carbone et al., 2004. |
| 39 | FAES0093 | AJ870444 | EST-SSR | GGA | Fvb4, 32 273 348 | Direct submission: Sargent, 2008. |
| 40 | FVES1392 | EX686688 | EST-SSR | AAAG | Fvb5, 10 543 285 | Rivarola et al., 2011. |
| 41 | FVES3693 | DV440263 | EST-SSR | AAC | Fvb5, 12 362 334 | Direct submission: Brese et al., 2005. |
| 42 | FVES2901 | EX664745 | EST-SSR | AGC | Fvb5, 19 192 042 | Rivarola et al., 2011. |
| 43 | FVES0233 | DY671012 | EST-SSR | AAG | Fvb5, 19 650 546 | Rivarola et al., 2011. |
| 44 | FVES1816 | DY667372 | EST-SSR | ACG | Fvb5, 20 772 009 | Rivarola et al., 2011. |
| 45 | FVES2349 | EX685812 | EST-SSR | GGC | Fvb5, 28 903 163 | Rivarola et al., 2011. |
| 46 | FVES3274 | DY674134 | EST-SSR | AGC | Fvb5, 3 549 108 | Rivarola et al., 2011. |
| 47 | FVES0794 | DV438342 | EST-SSR | AT | Fvb5, 3 629 957 | Direct submission: Brese et al., 2005. |
| 48 | FVES3770 | CX661464 | EST-SSR | AAC | Fvb5, 4 461 300 | Direct submission: Shulaev and Slovin, 2004. |
| 49 | FVES1470 | EX685374 | EST-SSR | AAG | Fvb5, 5 108 486 | Rivarola et al., 2011. |
| 50 | FAES0208 | CO380631 | EST-SSR | AAG | Fvb5, 6 479 121 | Direct submission: Carbone et al., 2004. |
| 51 | FVES2369 | EX684278 | EST-SSR | AAG | Fvb6, 12 584 411 | Rivarola et al., 2011. |
| 52 | FVES3346 | DY671621 | EST-SSR | AAG | Fvb6, 15 169 371 | Rivarola et al., 2011. |
| 53 | FVES2316 | EX686639 | EST-SSR | AAC | Fvb6, 16 869 389 | Rivarola et al., 2011. |
| 54 | FVES3440 | DY670464 | EST-SSR | AGC | Fvb6, 16 927 676 | Rivarola et al., 2011. |
| 55 | FVES0513 | DY668621 | EST-SSR | AAG | Fvb6, 17333025 | Rivarola et al., 2011. |
| 56 | FVES2533 | EX679315 | EST-SSR | GGC | Fvb6, 25 337 378 | Rivarola et al., 2011. |
| 57 | FVES0392 | DY675002 | EST-SSR | GGA | Fvb6, 32 768 853 | Rivarola et al., 2011. |
| 58 | FVES1640 | EX664885 | EST-SSR | AGC | Fvb6, 33 957 186 | Rivarola et al., 2011. |
| 59 | FVES1724 | DY674807 | EST-SSR | AG | Fvb6, 34 321 498 | Rivarola et al., 2011. |
| 60 | FVES0463 | EX672992 | EST-SSR | GGA | Fvb6, 36 900 824 | Rivarola et al., 2011. |
| 61 | FVES1070 | DY675299 | EST-SSR | GGC | Fvb6, 6 330 488 | Rivarola et al., 2011. |
| 62 | FAES0357 | CO381630 | EST-SSR | AC | Fvb7, 15 401 508 | Direct submission: Carbone et al., 2004. |
| 63 | FVES0634 | EX657120 | EST-SSR | GGT | Fvb7, 19 490 916 | Rivarola et al., 2011. |
| 64 | FAES0107 | CO380700 | EST-SSR | ATC | Fvb7, 20 925 390 | Direct submission: Carbone et al., 2004. |
| 65 | FVES0435 | DY669319 | EST-SSR | GGA | Fvb7, 22 962 133 | Rivarola et al., 2011. |
| 66 | FVES0128 | CX661870 | EST-SSR | AAC | Fvb7, 3 752 019 | Direct submission: Shulaev and Slovin, 2004. |
| 67 | FVES0381 | DY675732 | EST-SSR | AAG | Fvb7, 4684 340 | Rivarola et al., 2011. |
| 68 | RosCOS1371 | NA | EST-SSR | NA | NA | Cabrera et al., 2009. |

Acc. nr., GenBank accession number; Type, type of marker; Repeat, pattern of repeats; Chr., position, chromosome number and position; References.

Rousseau-Gueutin M, Richard L, Le Dantec L, Caron H, Denoyes-Rothan B. Development, mapping and transferability of Fragaria EST-SSRs within the Rosodae supertribe. Plant Breed. 2011 Apr 1;130(2):248–55.

Rivarola M, Chan AP, Liebke DE, Melake-Berhan A, Quan H, Cheung F, et al. Abiotic Stress-Related Expressed Sequence Tags from the Diploid Strawberry Fragaria vesca f. semperflorens. Plant Genome. 2011 Mar 1;4(1):12–23.

Cabrera A, Kozik A, Howad W, Arus P, Iezzoni AF, van der Knaap E. Development and bin mapping of a Rosaceae Conserved Ortholog Set (COS) of markers. BMC Genomics. 2009;10(1):562.
