# Supplementary material for: Population genetic analysis of a global collection of Fragaria vesca using microsatellite markers
Source: PLoS One. 2017 Aug 30;12(8):e0183384. doi: 10.1371/journal.pone.0183384 (PMC5576660; doi:10.1371/journal.pone.0183384)
Supplement: S3 Table — Nei’s genetic distance (below diagonal line) and pairwise population Fst values (above diagonal line) between groups. (DOCX) [file pone.0183384.s005.docx]

**S3 Table. Nei’s genetic distance and pairwise population F_st_ values.**

|  | *F. vesca* subsp. *americana* | *F. vesca* subsp. *bracteata* ‘Rocky Mts.’ | *F. vesca* subsp. *bracteata* ‘Pacific’ | *F. vesca* subsp. *californica* | Cultivars | Eurasia w/o Iceland | *F. chinensis* | Iceland | Japan | American *F. vesca* subsp. *vesca* | *F. viridis* |
| --- | --- | --- | --- | --- | --- | --- | --- | --- | --- | --- | --- |
| *F. vesca* subsp. *americana* | - | 0.214 | 0.234 | 0.399 | 0.349 | 0.269 | 0.777 | 0.347 | 0.464 | 0.277 | 0.668 |
| *F. vesca* subsp. *bracteata* ‘Rocky Mts. | 0.146 | - | 0.119 | 0.260 | 0.256 | 0.194 | 0.690 | 0.253 | 0.310 | 0.186 | 0.571 |
| *F. vesca* subsp. *bracteata* ‘Pacific’ | 0.144 | 0.104 | - | 0.302 | 0.320 | 0.246 | 0.772 | 0.307 | 0.378 | 0.259 | 0.663 |
| *F. vesca* subsp. *californica* | 0.168 | 0.147 | 0.135 | - | 0.454 | 0.354 | 0.830 | 0.434 | 0.610 | 0.353 | 0.711 |
| Cultivars | 0.193 | 0.184 | 0.198 | 0.211 | - | 0.071 | 0.760 | 0.062 | 0.164 | 0.087 | 0.660 |
| Eurasia w/o Iceland | 0.179 | 0.170 | 0.187 | 0.204 | 0.034 | - | 0.690 | 0.055 | 0.127 | 0.045 | 0.566 |
| *F. chinensis* | 0.583 | 0.564 | 0.571 | 0.601 | 0.547 | 0.559 | - | 0.752 | 0.860 | 0.743 | 0.808 |
| Iceland | 0.196 | 0.190 | 0.203 | 0.220 | 0.025 | 0.026 | 0.547 | - | 0.181 | 0.085 | 0.663 |
| Japan | 0.193 | 0.186 | 0.187 | 0.220 | 0.037 | 0.053 | 0.543 | 0.047 | - | 0.163 | 0.745 |
| American *F. vesca* subsp. *vesca* | 0.143 | 0.144 | 0.165 | 0.161 | 0.040 | 0.036 | 0.547 | 0.051 | 0.062 | - | 0.585 |
| *F. viridis* | 0.459 | 0.464 | 0.476 | 0.489 | 0.425 | 0.405 | 0.609 | 0.435 | 0.427 | 0.404 | - |

Nei’s genetic distance between groups is shown below the diagonal line with the pairwise population F_st_ values between groups shown above the diagonal line.
